# Targeting the retinoic acid signaling pathway as a modern precision therapy against cancers

**DOI:** 10.3389/fcell.2023.1254612

**Published:** 2023-08-11

**Authors:** Kousalya Lavudi, Shreya Madhav Nuguri, Zianne Olverson, Anantha Krishna Dhanabalan, Srinivas Patnaik, Rekha Rani Kokkanti

**Affiliations:** ^1^ Department of Radiation Oncology, College of Medicine, The Ohio State University, Columbus, OH, United States; ^2^ Comprehensive Cancer Center, The Ohio State University, Columbus, OH, United States; ^3^ School of Biotechnology, KIIT University, Bhubaneswar, Odisha, India; ^4^ Department of Food Science and Technology, The Ohio State University, Columbus, OH, United States; ^5^ Centre for Advanced Study in Crystallography and Biophysics, University of Madras, Guindy Campus, Chennai, India; ^6^ Department of Biotechnology, Sri Padmavati Mahila Visvavidyalayam, Tirupati, Andhra Pradesh, India

**Keywords:** retinoic acid, aldehyde dehydrogenases, CYP26A1, cancer cell proliferation, tumor relapse, immune checkpoint inhibitors, chemoresistance

## Abstract

Retinoic acid (RA) is a vital metabolite derived from vitamin A. RA plays a prominent role during development, which helps in embryological advancement and cellular differentiation. Mechanistically, RA binds to its definite nuclear receptors including the retinoic acid receptor and retinoid X receptor, thus triggering gene transcription and further consequences in gene regulation. This functional heterodimer activation later results in gene activation/inactivation. Several reports have been published related to the detailed embryonic and developmental role of retinoic acids and as an anti-cancer drug for specific cancers, including acute promyelocytic leukemia, breast cancer, and prostate cancer. Nonetheless, the other side of all-trans retinoic acid (ATRA) has not been explored widely yet. In this review, we focused on the role of the RA pathway and its downstream gene activation in relation to cancer progression. Furthermore, we explored the ways of targeting the retinoic acid pathway by focusing on the dual role of aldehyde dehydrogenase (ALDH) family enzymes. Combination strategies by combining RA targets with ALDH-specific targets make the tumor cells sensitive to the treatment and improve the progression-free survival of the patients. In addition to the genomic effects of ATRA, we also highlighted the role of ATRA in non-canonical mechanisms as an immune checkpoint inhibitor, thus targeting the immune oncological perspective of cancer treatments in the current era. The role of ATRA in activating independent mechanisms is also explained in this review. This review also highlights the current clinical trials of ATRA in combination with other chemotherapeutic drugs and explains the future directional insights related to ATRA usage.

## 1 Introduction

Retinoic acids (RAs), which are considered the active metabolites of vitamin A, play a major role in cellular differentiation and organ development (Cunningham and Duester, 2015). RA binds to its receptors and activates downstream gene transcriptional activity, resulting in the expression of respective gene targets and their signaling mechanisms including cell differentiation, proliferation, and apoptosis. Balmer and Blomhoff (2002) showed that more than 532 genes are considered the regulatory targets of retinoic acids in both direct and indirect ways. Approximately 193 human genes act as the direct targets to the retinoic acid receptor/rexinoic acid receptor (RAR/RXR) heterodimers (Hosoda et al., 2015).

RA, particularly all-trans retinoic acid (ATRA), which is the active form of vitamin A, is involved in a wide range of biological processes, including embryogenesis, cell differentiation, and apoptosis (Kanungo, 2017; Tagliaferri et al., 2020; Brown, 2023). RA has been traditionally recognized as a molecule that induces cell differentiation and prevents the proliferation of progenitor cells (Díez-del Val and Martínez-Blázquez, 2003). However, recent research has expanded our understanding of the functions of RA, revealing its involvement in promoting cellular proliferation, inducing stemness, and regulating progenitor cell behavior (Malta et al., 2018; Mezquita et al., 2018; Mishra et al., 2018). RA has been extensively studied in the context of cancer progression; while it is known to induce cell differentiation in experimental models and has been used effectively in the treatment of acute promyelocytic leukemia (APL), it has also been found to induce stemness and progenitor cell proliferation in different cancer cell types. The effects of RA on cancer cells can be both protumorigenic and anti-tumorigenic, thus highlighting its complex role in cancer development (Marcato et al., 2015).

### 1.1 Synthesis of retinoic acids

**FIGURE 1:**
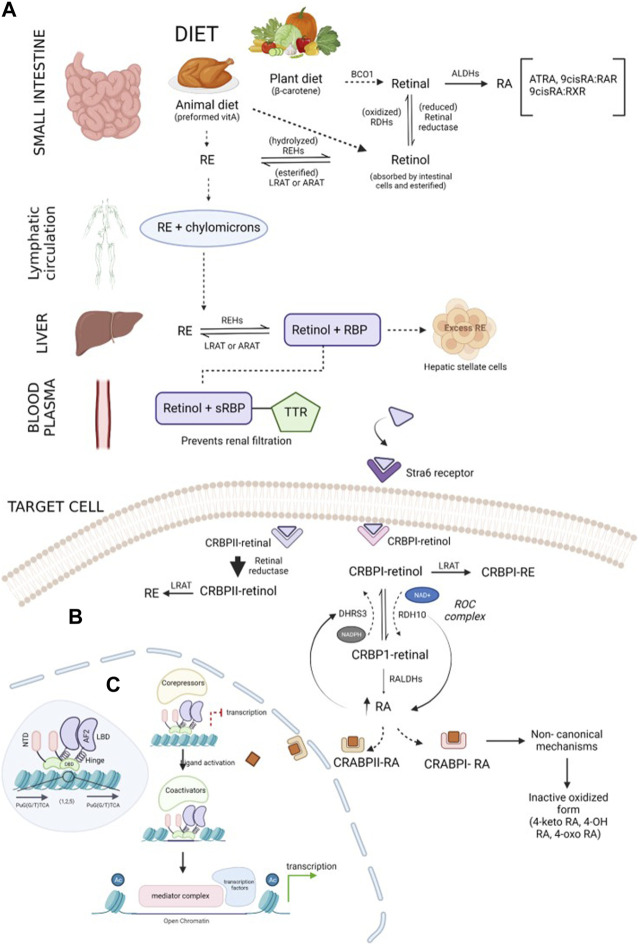
Schematic representation of the retinoid synthesis pathway: **(A)** vitamin A is converted to its active forms (RE and retinol) in the small intestine. β-Carotene obtained through plant diet is acted upon by the BCO1 enzyme, forming retinal molecules. Retinal can either be reduced to retinol by retinal reductase or oxidized by ALDHs, forming RA. Preformed vitamin A, retinol, and RE obtained through animal diet can be interconverted by enzymes REHs, LRAT, or ARAT. Chylomicrons transport RE through the lymphatic system and transfer it to the liver. The required RE is converted into retinol, and excess RE is stored in hepatic stellate cells. Lipophilic retinol binds to the retinol-binding protein to traverse in an aqueous medium. In blood plasma, the retinol–RBP complex tethers to TTR, preventing renal purification by increasing the compound weight. Upon reaching the target cell, TTR shreds from the complex, and sRBP delivers retinol to the Stra6 receptor present in the plasma membrane of the cell. **(B)** Regulation of retinoids inside the target cell: retinol is received by binding proteins (CRBPI and II) present in the cytosol. CRBPI-binding proteins facilitate the intake of retinol by the conversion of retinol to RE or RA. Based on the RA requirement of the cell, CRBPI-bound retinol is oxidized to CRBPI-bound retinal by RDHs and ROC, which are, in turn, oxidized by RALDHs, forming RA. A negative feedback loop regulates RA levels in the cells through the ROC complex. The ROC heterooligomeric compound consists of DHRS3 (reductive action using the NADPH cofactor) and RDH10 (oxidative action using the NAD^+^ cofactor). An increase in RA upregulates DHRS3 control and downregulates RDH10 activity, and a decrease in RA follows *vice versa* conversion. RA bound to CRABPI undergoes non-canonical mechanisms, followed by conversion to its inactive oxidized form and elimination, while RA bound to CRABPII is transported to RA receptors in the nucleus to activate transcription of target genes. Excess RA formation is toxic and is prevented by the CRABPII protein. The retinal bound to CRABPII has a higher reduction rate than its oxidative tendency. **(C)** Overview of the transcription mechanism of target genes by the RA receptor: RA receptors are present in the heterodimerized form bound to target response elements composed of direct repeats of PuG(G/T)TCA (shown in the blue-shaded region inside the nucleus). The receptor is composed of five main domains (NTD, DBD, hinge, LBD, and F-region). The unliganded receptor recruits corepressors which repress the transcription of the genes. CRABPII delivers the ligand to the receptor. CRABPII is sent back to the cytosol, followed by the short interaction of the binding protein with the RA receptor. Upon ligand activation by RA, the corepressors disassociate, promoting chromatin decompaction by coactivators. In the third step, the coactivators disassemble, followed by assembling of the mediator complex, which further recruits transcription machinery, initiating the expression of target genes.

Vitamin A is an essential fat-soluble micronutrient, with recommended dietary allowances ranging from 400 to 700 mcg RAE (retinol activity equivalents) (Institute of Medicine. Food and Nutrition Board, 1998). It is obtained as preformed retinoids (retinol and retinyl esters (REs)) from animal sources (liver, egg yolk, and milk) and as pro-vitamin A (β-carotenoids) from plant sources (green, red, or orange fruits and vegetables) (Siddikuzzaman et al., 2011; Gilbert, 2013; McEldrew et al., 2023). These ingested retinoid forms are metabolized to retinol and retinoic acid after absorption to perform biological functions (McEldrew et al., 2023). Retinyl esters obtained from meat are hydrolyzed by lipase in the upper part of the small intestine, forming retinol (Institute of Medicine. Food and Nutrition Board, 1998), while BCO1 (β-carotene 15-15’ oxygenase) cleaves β-carotene from plant diet to form retinaldehyde (Figure 1A). This retinaldehyde can be irreversibly oxidized to retinoic acids via retinaldehyde dehydrogenases, also called aldehyde dehydrogenases (ALDHs), or reversibly reduced to retinol by retinal reductase (Blaner et al., 2016). The retinol acquired through diet is absorbed by the intestinal cells and esterified under the actions of lecithin:retinol acyltransferase (LRAT) or acyl-CoA:retinol acyltransferase (ARAT) to RE which subsequently combines with chylomicrons and is transported to the liver through lymphatic circulation (Blaner et al., 2016; Ni et al., 2019; Jin et al., 2022). In the liver, RE is hydrolyzed by retinyl ester hydrolases (REHs) to retinol and binds to retinol-binding protein 4 (RBP4), forming a complex (Goodman, 1984; Ni et al., 2019). The retinol–RBP complex binds with transthyretin (TTR) in blood, which increases the molecular weight and prevents retinol loss during renal filtration (Jin et al., 2022). The retinol–RBP–TTR complex circulates in blood until it reaches the target cells, where its uptake is facilitated by the signaling receptor and transporter of the retinol STRA6 (Stra6) receptor (Ni et al., 2019; Liang et al., 2021; Jin et al., 2022). In the cells, retinol is oxidized to retinaldehyde via the actions of retinol dehydrogenases (RDHs), and it further oxidizes to form retinoic acids (Ni et al., 2019). Excessive retinoids are stored in hepatic stellate cells in the form of RE (Jin et al. 2022). Intracellular ATRA is balanced by synthesis and degradation processes regulated via cytochrome P450 species. Intracellular ATRA binds to cellular RA-binding protein (CRABP1), which oxidizes ATRA to inactive oxidized retinoids (4-keto RA, 4-oxo-RA, 18-hydroxy RA, and 4-hydroxy RA) (Napoli et al., 1995; Jiao et al., 2020).

Structurally, retinoids are constructed by three main units: a cyclic hydrophobic group at the head portion, a polar terminus at the tail portion, and a polyene chain linking the two units (Liang et al., 2021; Jin et al., 2022). They are broadly classified as natural endogenous retinoids (ATRA and isomers 9-cis-retinoic acid and 13-cis-retinoic acid) and synthetic retinoids (e.g., UAB30, peretinoin, bexarotene, ST1926, and WYC-209). Synthetic retinoids are more stable and have low toxicity, high selectivity, and high effectiveness compared to natural derivatives (Jin et al., 2022). Two ATRA-dependent nuclear retinoid receptors involved are the retinoic acid receptor (RAR) and retinoid X receptor (RXR). ATRA isomer 9-cis-RA has a high affinity for RXRs and RARs (Liang et al., 2021), while 13-cis-RA has low affinity toward the receptors although they act as agonists of RXR and RAR (Layton, 2009).

### 1.2 Regulation of ATRA in cells

Multiple binding proteins and receptors are involved in the transportation and regulation of retinoids in serum and intracellular space (Napoli, 2016; Belyaeva et al., 2019; Nhieu et al., 2022). Serum retinol-binding protein (sRBP), encoded by the gene *Rbp4*, helps lipophilic retinol to move through the aqueous phase. The retinol enters the cell via the sRBP receptor, Stra6, located in the plasma membrane (Figure 1B). CRBP1, a major intracellular retinol-binding protein, facilitates the cellular uptake of retinol by Stra6 and provides a substrate for enzymes generating RE or RA (Napoli, 2016). Studies suggest that the cellular uptake of retinol is related to RE formation (LRAT catalyzed) and CRBP1 concentration; retinol uptake linearly increases until CRBP1 concentration is reached and is inversely affected by inhibiting RE (Amengual et al., 2014; Napoli, 2016). Tissue-specific effects have been observed by retinol-esterifying enzymes (LRAT and ARAT) in maintaining retinoid homeostasis. Additionally, by binding to retinol, CRBP1 prevents the exposure of “free retinol” to catabolism, excess RA biosynthesis, which may constitute retinoid toxicity, or other opportunistic metabolisms, such as xenobiotic-metabolizing enzymes (Institute of Medicine. Food and Nutrition Board, 1998). The flux between retinol and RE has been indicated by the holo-CRBP1/apo-CRBP1 ratio (Napoli, 2016). Retinal produced by the cleavage of carotenoids may undergo reduction to retinol, subsequently forming RE, or oxidation to retinaldehyde, forming RA (RDH and RALDH catalyzed). Dietary carotene is not harmful, although excess RA is toxic (Napoli, 2016). This excess RA biosynthesis is prevented by CRBP2; the oxidation rate of CRBP2-bound retinol is 300 times less than the reduction rate to retinol, allowing RE conservation and transportation through chylomicrons (Napoli, 2016).

Two groups of the short-chain dehydrogenase/reductase (SDR) superfamily participate in RA homeostasis regulation: SDR16C (dehydrogenase/reductase3 (DHRS3) and retinol dehydrogenase 10 (RDH10)) and SDR7C. Most SDR16C members (except DHRS3) have higher affinity toward the NAD(H) cofactor serving in the oxidative direction, while SDR7C prefers the NADP(H) cofactor (Belyaeva et al., 2019). RDH10 is a NAD(+)-dependent retinol dehydrogenase that catalyzes retinol to retinaldehyde. DHRS3 has little-to-no activity; although when co-expressed with RDH10, it exhibits strong retinaldehyde reductase activity (Veech et al., 1969). Studies indicate that RDH10 and DHRS3 bind to form a heterooligomeric retinoid oxidoreductive complex (ROC) that modulates the interconversion of retinol and retinal using NAD(H) and NADPH cofactors (Belyaeva et al., 2019). The concentration of RA levels is regulated by a negative feedback loop monitored by the ROC complex. An increase in RA is followed by the upregulation of DHRS3 activity and a decrease in RDH10 activity and *vice versa* (Belyaeva et al., 2019). In addition to ROC, several RDHs (RDH10, RDHE2S, and RDHE2) irreversibly oxidize retinol. Three types of cytosolic RALDH (RALDH1, 2, and 3) catalyze the irreversible oxidation of retinaldehyde to RA. Again, the binding protein, CRBP1, plays an important role in channeling retinal through aqueous media, from the microsome to the cytosol, via the action of the RALDH enzyme. Like CRBP, RA binds to proteins such as CRABP1 and 2 and fatty acid-binding protein 5 (FABP5), which are responsible for performing diverse functions. CRABP1 provides a substrate for RA metabolism via CYP-450 enzymes. CRBP2 is mainly responsible for the translocation of RA from the cytosol to the nucleus and delivering the ligand to RAR for transcriptional activity, while FABP5 delivers RA to PPARδ/β (Napoli, 2016; Belyaeva et al., 2019). These RAR-mediated activities trans-regulate transcriptions of various genes in the nucleus, termed as canonical mechanisms of RA (Nhieu et al., 2022). Additionally, the RA-binding proteins act as RA-mediated signalosomes in a cell context-dependent manner (Nhieu et al., 2022) and moderate non-genomic or noncanonical activities of RA occurring in the cytosol with no alteration in gene expression (Napoli, 2016).

**FIGURE 2:**
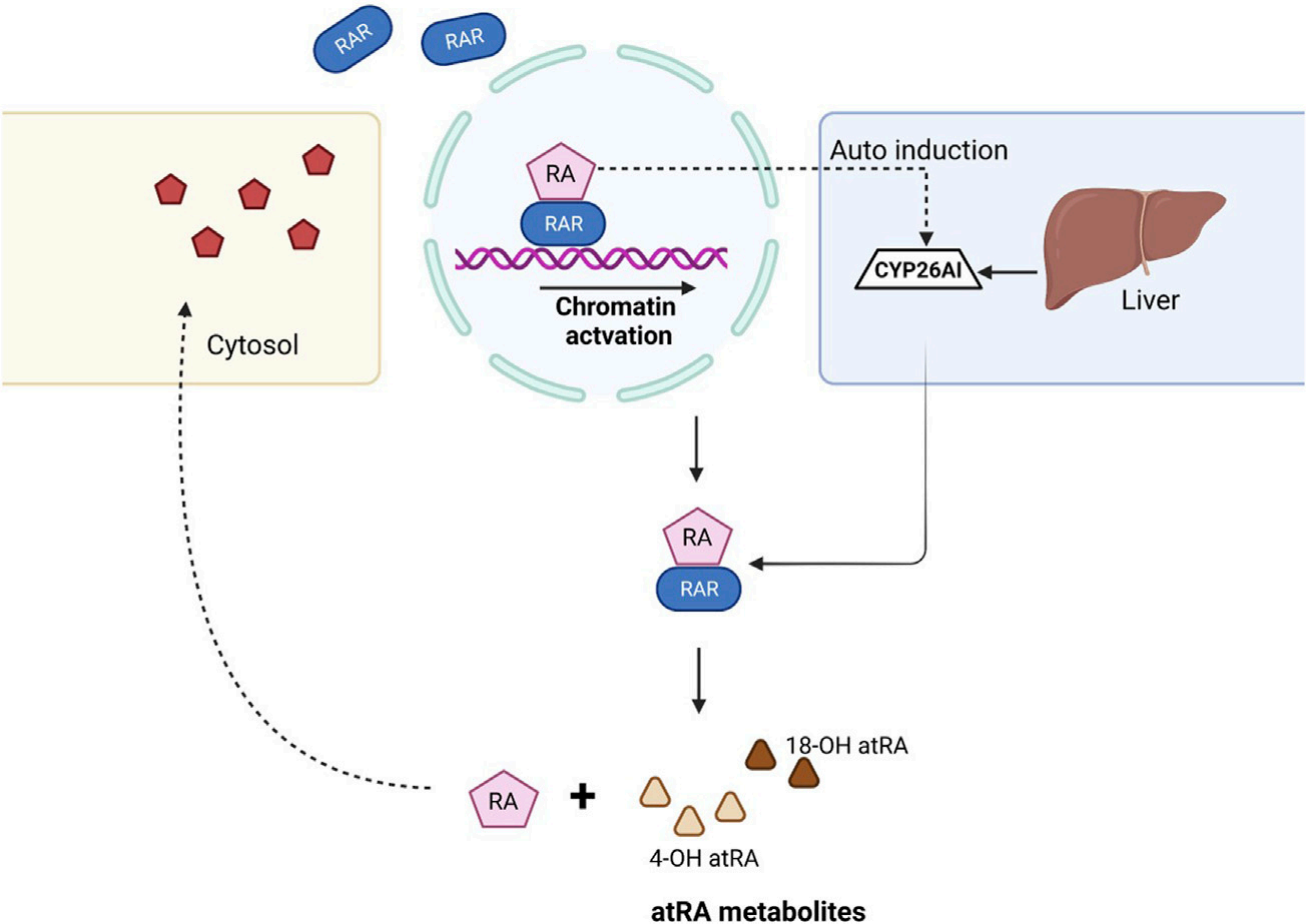
Mechanism of ATRA clearance in the cell. When the RA ligand binds to the RAR, gene transcription takes place and results in downstream activation. Once the RA role is fulfilled, CYP26 family enzymes auto-induce from the liver and help in the clearance of ATRA, thus balancing the levels of RA within the cell. ATRA, with the help of CYP26A1 and CYP26B1, dissociates to form 4-OH atRA and 18-OH atRA.

Excess ATRA within the cells results in detrimental effects during the developmental stages and is required to undergo degradation. The cytochrome P450 family plays a crucial role in the maintenance of endogenous ATRA levels, and mutations in these genes resulted in teratogenic effects (Topletz et al., 2015). Several studies confirmed that the biological activity of ATRA within the cell is confined to ∼1 h and later undergoes the clearance process. CYP26A1, CYP26B1, and CYP26C1 play a major role in ATRA clearance. CYP26A1 and CYP26B1 hydroxylate ATRA to 4-OH atRA and 18-OH atRA, respectively, which are considered primary metabolites of ATRA and are hydroxylated further (Stevison et al., 2015). The detailed mechanism of the biochemical and physiological importance of ATRA was explained previously (Isoherranen and Zhong, 2019) (Figure 2).

### 1.3 Retinoid receptors

The retinoic acid receptor belongs to the class of RXR heterodimers, a subcategory of the transcription factor group called the nuclear receptor (NR) superfamily, which plays a crucial role in many activities, including cell proliferation, apoptosis, and metabolism. Regardless of the variations in structural and functional characteristics of NR subgroups, their architecture remains similar. They are composed of five major domains (A–E), namely, a disordered N-terminal domain (NTD), which contains a ligand-independent activator function-1 (AF-1) region that binds to co-regulatory proteins; a conserved DNA-binding domain (DBD) that secures specific DNA sequences; a hinge region which confers the rotation of the DBD and LBD, overcoming steric hindrance between them; and a second most conserved ligand-binding domain (LBD), which offers a ligand-hooking region (Bastien and Rochette-Egly, 2004; Weikum et al., 2018; Porter et al., 2019). RA signaling is directed by RAR and RXR; RXR can self-dimerize to exhibit its activity or heterodimerize with RAR, forming the RXR/RAR complex (Di Masi et al., 2015). Each of these receptors have three isotypes (α, β, and γ) represented by different genes (Leid et al., 1992; Mangelsdorf and Evans, 1995). Ligands ATRA and 9-cis RA induce activation in RARs, while 9-cis RA initiates RXRs. The transcription mechanism of target genes by NR receptors was reviewed in detail by Napoli (2016). Prior to ligand introduction, these receptors are bound to target DNA promoters in the nucleus (Figure 1C) called RA response elements (RARE). RARE generally consist of direct repetition (DR) of two core motifs, PuG(G/T)TCA, either separated by five (DR5), two (DR2), or one (DR1) base pair (Leid et al., 1992; Bastien and Rochette-Egly, 2004). DBDs in the dimerized receptors recognize specific response elements, and based on DR spacing, they develop a dimerization interface to increase the RARE binding efficiency (Bastien and Rochette-Egly, 2004). Unliganded, DNA-bound RA receptors recruit repression cofactors (NCoR, SMRT, and HDACs) that condense chromatin, obstructing the transcription of the target genes. In the cytosol, RA binds to cellular retinoic acid-binding protein II (CRABPII) that delivers the retinoid ligand to the nucleus (Dong et al., 1999; Thatcher and Isoherranen, 2009). Ligand activation leads to a conformational change which sheds the co-repressors and forms a hydrophobic surface for coactivators (SRC/p160 family, p300/CBP, and CARM-I) to tether. These coactivators recruited by the synergy of AF-1 and AF-2 of the dimerized receptors decompact the chromatin structure through histone acetyltransferase activity (HAT) (Kundu and Das, 2022) and ATP-dependent remodeling, weakening the linkage between N-terminal histone and nucleosome DNA. In the RAR/RXR heterodimer, RAR is dominant in recruiting co-activators as liganded RXR alone cannot disassociate corepressors. However, with liganded RAR, there is a synergy between the receptors through the AF-1 and AF-2 domains that induces the cooperative binding of coactivators to the heterodimer (Willy and Mangelsdorf, 1998; Germain et al., 2002; Bastien and Rochette-Egly, 2004). After decompactation, the co-activators undergo acetylation, decreasing their interaction with the RA receptor, and eventually dissociate; the receptors subsequently assemble the SMCC mediator complex. This complex promotes the entry of transcription machinery (general transcription factors, RNA pol II), promoting the transcription of the target gene. The RA receptors are activated by the phosphorylation of different signaling pathways (MAPK, PKA, and Akt). This stimulation regulates the recruitment of various co-activators, co-repressors, and transcription factors, which can, in turn, be phosphorylated to assemble more co-factors. The extent of RA-mediated transcription is controlled by the ubiquitin–proteasome pathway; however, its activity on the retinoid transcription mechanism is complex. The receptor isotypes are not regulated similarly by the pathway (Kopf et al., 2000); in the RARγ/RXR complex, ubiquitin–proteasome plays a dual role in promoting and degrading transcription machinery, while in RARα/RXR, the inhibition of the proteasome has been associated with increased transcription (Bastien and Rochette-Egly, 2004). The system inhibits transcription mainly by degrading transcriptional activators and/or the RAR/RXR heterodimer receptors. During degradation, RAR and the activator proteins are multi-ubiquitinated and targeted by the 26S proteasome for cleavage or by proteasome systems, such as SUG-1, recruited at the AF-2 domain of the receptor. In addition to protein degradation, the importance of the ubiquitin–proteasome pathway in RA-mediated transactivation, particularly in the case of RARγ, was demonstrated by Giannì et al. (2002). An elaborate mechanism of this binary role is currently unknown although several studies have demonstrated the involvement of ubiquitin ligases in the polymerase II transcriptional complex (Varkey, 2021) and the association between proteasome SUG-I and general transcription factor TFIIH (GTF TFIIH) (Weeda et al., 1997). Furthermore, Gonzalez et al. (2002) defined a non-proteolytic role of the 19S subcomplex in efficient elongation of RNA polymerase II during transcription. Kinyamu et al. (2005) critically examined the role of the ubiquitin–proteasome pathway in histone modification during chromatin remodeling.

In addition to being involved in the expression of retinoid-target genes, the retinoids can transactivate or antagonize the activation of several pathways, including AP-I activity, Smad-regulated genes, and PI3/Akt pathway (Bastien and Rochette-Egly, 2004) through cross-signaling. Thus, an off-track of any factor or signaling pathway may lead to the occurrence of disease. A prominently studied disease is acute promyelocytic leukemia (APL), which is caused by the fusion genes PML-RARα and PLZF-RARα, which results in the atypical selection of corepressors suppressing the RA-targeted genes responsible for the terminal differentiation of promyelocytes (Altucci and Gronemeyer, 2001). Studies show that RAR activity is not required for normal hematopoiesis although these receptors may play a significant role in modifying or regulating a variety of myeloid progenitors (Collins, 2002). In addition, a number of cancers have been linked to the loss of RARβ2 expression, which is regarded as a tumor suppressor, although the genes regulated by it have not been comprehensively studied (Bastien and Rochette-Egly, 2004).

## 2 Retinoic acid and its signaling mechanism

Retinoic acids, which are highly considered dietary regulators, participate in various signaling mechanisms to maintain the biological needs within the cell. RAs, via RARs, cross the nuclear membrane and bind to the RARE sequences and are involved in the gene regulation mechanism. Several studies showed that genes, which are involved in cell regulation, proliferation, repair, and maintenance, have a conserved RARE domain within the promoter, thus activating gene transcription. This causes the transcriptional activation of several genes which are involved in proliferative mechanisms (Dong et al., 2003; Ablain and de Thé, 2014; Quintero Barceinas et al., 2015). Some studies also showed that this mechanism is involved in acquiring resistance in various cancers, including osteosarcoma (Lu et al., 2017; Shi et al., 2017; Li et al., 2022). The detailed signaling mechanism is explained in the previous section.

### 2.1 Gene development by retinoic acids

Considering RA a key regulator in the developmental process, RAs have gained significant attention in gene development. The availability of ATRA is highly essential for gene activation and regulation mechanisms for embryonic developmental activity. The existence of the RARE sequence in these genes makes them dependent on ATRA availability to perform specific functions. Cunningham and Duester (2015) elaborately explained the mechanism regarding RA signaling and its role in organ and limb development.

Several reports confirmed the vital role of RA in developmental stages as a morphogen that induces differentiation. However, recent studies by Iturbide et al. (2021) confirmed that retinoic acids play a role in the totipotency window and are derived from pluripotent embryonic stem cells at the blastomere stage. They named this group “2-cell-like” cells. According to their studies, the authors confirmed that the maximal induction of 2CLC was observed at a concentration of 0.53 uM, and the induction gradually decreases at higher concentrations. This study clearly explains the canonical role of RA in developmental processes.

### 2.2 Cell differentiation

**FIGURE 3:**
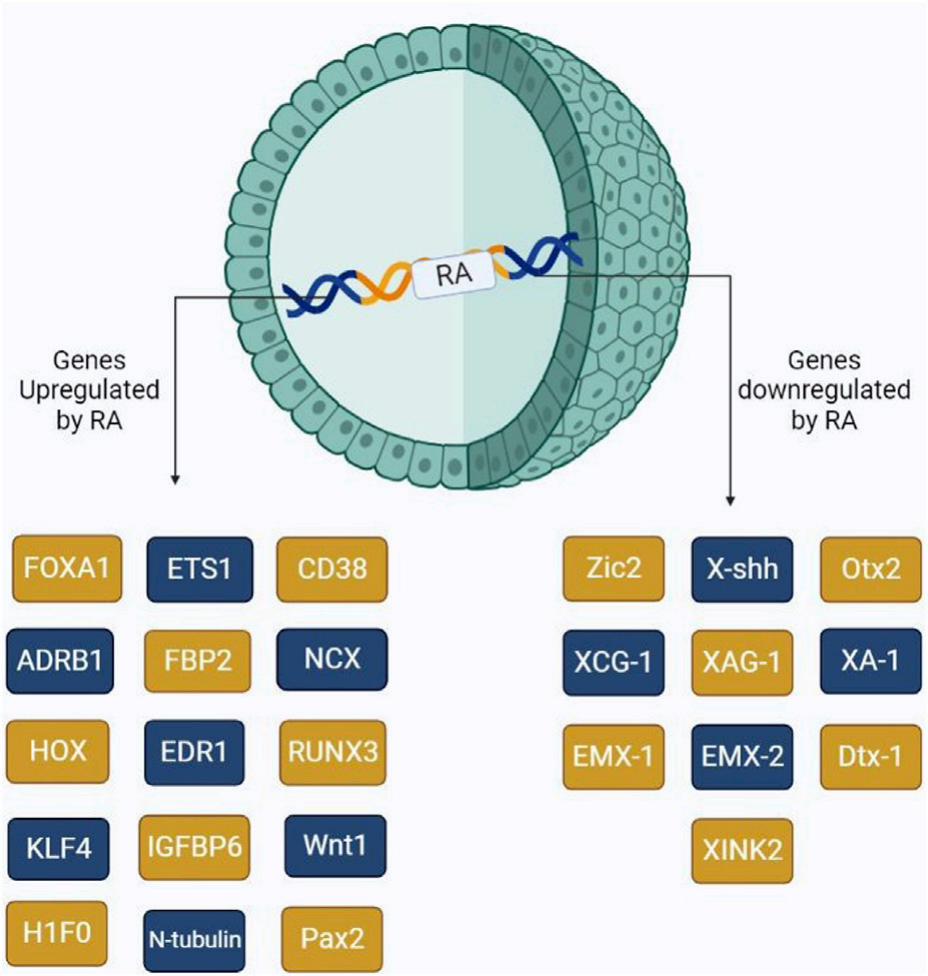
Schematic representation of the major genes activated by the presence of RA. RA upon binding to its receptors, RAR and RXR, triggers the activation of several genes including those involved in cell differentiation and cancer cell proliferation.

RAs have been extensively studied to understand their collective roles including cell differentiation at very early stages. Other reports have shown that RA might induce cancer cell development, thus resulting in progression. Several genes, which are involved in differentiation, are directly under the control of RA availability. These genes include ETS1 (Raouf et al., 2000), CD38, FOXA-1, H1F0, HOXA1, ADRB1, EDR1, FBP2, IGFBP6, NCX, and RUNX3. ETS1 is a proto-oncogenic factor that plays a role in the proliferation of cancer cells. Lower RA levels in the cells result in the oncolytic activity of ETS1, thus resulting in tumor proliferation via the MAPK signaling mechanism, which allows for the regulation of matrix metalloproteases. ETS1 as a proto-oncogene was explained by Dittmer (2003). On the other hand, FOXA1, also controlled by RA, plays a major role in cell differentiation. However, studies show that increased FOXA1 expression is evident in prostate, ovarian, and breast cancer progression. Hua et al. (2009) showed that the co-evolvement of binding sites between RAR, FOXA1, and ERα results in the overexpression of Erα, consequently provoking the Erα expression, resulting in tumor progression. RUNX3, which also plays a key role in cell differentiation, is involved in pancreatic cancer progression by upregulating various signaling mechanisms, including Wnt, TGF-β, and Hippo-YAP (Chuang et al., 2023). HOXA1 is mainly known to be involved in cell differentiation, growth, apoptosis, metastasis, and regulation of embryonic development. Dysregulation of this gene primarily involves tumor development in oral, melanoma, gastric, and bladder cancers (Chen et al., 2022). Figure 3 shows the major genes upregulated/downregulated by RA gene activation.

### 2.3 Epigenetic regulation of RA and its receptors in genome maintenance and cancer development

Retinoic acids as morphogens play a key role in gene regulation and development. In the absence of RA, during embryonic stem cell development, enhancers are ideally silenced by histone deacetylases (HDACs) 1, 2, and 3 at the RARE sequences in RARβ, HOXA1, and CYP26A1 (Urvalek and Gudas, 2014). Nonetheless, the presence of RA in the cell helps in converting the closed chromatin complex to the open form by acetylation using HAT at HEK4, HEK9, and HEK27 regions, leading to downstream transcriptional activation. This mechanism is considered the age-old mechanism in the eukaryotic cells in both development and regulation.

#### 2.3.1 Hypermethylation of RARβ receptors and cancer progression

RARs, including RARα, RARβ, and RARγ, play a critical role in gene regulation; however, expression of these receptors highly correlates with chromatin remodeling. Several studies have shown that the RARβ2 subunit is mainly involved in the differentiation mechanism, and this receptor expresses its activity in the acetylated form. Inactivation of RARβ2 results in tumor proliferation and cancer progression. Sarkar et al. (2015) validated the correlation between the RARβ2 gene and tumor progression regarding chromatin activation. More than 70% of cancers show inactivated RAR 2 gene function. In melanoma, RARβ2 silencing is due to hypoacetylation of the gene rather than hypermethylation. CpG island methylation of RARβ led to the methylation of this gene, causing subsequent non-functionality in cervical cancer patients (Wongwarangkana et al., 2018).

#### 2.3.2 Role of RARα in epigenetic regulation with relation to cancer progression

RARα plays a dual role in cells with relation to RA availability. When RA is available, binding of RA to RARα results in downstream gene activation and controls the cell differentiation mechanism. However, when RA is unavailable, or cells become resistant to RA, RARα activation occurs via Erα receptors, thus triggering cell proliferation and tumor metastasis in breast cancer progression (Rossetti et al., 2016).

Retinoic acid modulates gene transcription by binding to RARs at a specific sequence called RARE and results in epigenetic modifications. RARs act as a molecular switch. When ligands are unavailable, RAR-RXR heterodimers bind to NcoR/SMRT complexes and recruit HDAC, along with polycomb repressive complex 2 (PRC2). This results in the trimethylation of H3 at Lys27, which eventually leads to closed chromatin complex formation and gene silencing (Glass and Rosenfeld, 2000; Jepsen et al., 2000). Upon ligand availability and binding to the RAR-RXR heterodimer, conformational alterations are observed in DBD and recruit activator complexes, including NCoA1, NCoA2, and NCoA3. This complex further replaces HDAC with HATs and regulates histone H3 lysine 4 methylation, ultimately resulting in the opening of the chromatin complex, which further results in activation (Ozgun et al., 2020) (Figure 1C).

#### 2.3.3 Retinoic acid in cancer stem cell differentiation

In addition to its effects on cancer cells, RA can also influence the tumor microenvironment (TME). It can shape the tumor stromal cells, favoring tumor growth and invasion (Liu et al., 2011). Furthermore, RA plays a role in tumor immunosuppression, decreasing immature myeloid cells and inducing regulatory T cells (Bazewicz et al., 2019). The role of RA in mammary gland development and breast cancer is of particular interest (Cho et al., 2012). It can exert pro-differentiation and antidifferentiation effects during mammary gland development and breast cancer progression (Rossetti et al., 2016; Berardi et al., 2021). RA can also exert extra-genomic actions and regulate the expression of specific genes (Cecile, 2015; Zhang et al., 2015). Its impact on ALDH1 expression has been observed in ovarian cancer cells, where it decreases ALDH1 expression and inhibits tumor formation and invasive properties (Young et al., 2015).

Recent studies have shown that RA induces proliferation in early neurogenesis in the developing mouse cerebral cortex and adult hippocampus (Mosher and Schaffer, 2018). Additionally, it was found that in stem-like glioma cells, RA promotes proliferation (Choschzick et al., 2014; Haushalter et al., 2017; Mishra et al., 2018). In the context of hematopoietic stem cells, it was shown that RA prevents the differentiation of dormant primitive hematopoietic stem cells and instead induces the differentiation of more mature blood cells. This suggests that RA plays a role in regulating the balance between stem cell self-renewal and differentiation in the hematopoietic system (Masetti et al., 2012). Furthermore, it was found that during spermatogenesis, RA induces both cell differentiation and proliferation, contributing to the development of mature sperm cells (Endo et al., 2017).

The expression of RARs and RXRs is often dysregulated in various cancer types. The overexpression and activation of RARs have been implicated in the inappropriate behavior of cancer stem cells (CSCs) in malignancies such as AML, cholangiocarcinoma, colorectal cancer, clear cell renal cell carcinoma, hepatocellular carcinoma, prostate cancer, pancreatic ductal adenocarcinoma, and ovarian cancer (Bleul et al., 2015; Herreros-Villanueva et al., 2015; Guo et al., 2016; Wang et al., 2016; Fettig et al., 2017; Mezquita et al., 2018; Fan et al., 2021). The precise mechanisms underlying the role of RA in tissue-specific stem cells and the development of cancer stem cells are not fully understood yet and require further investigation.

### 2.4 Retinoic acids and their feedback mechanisms

Retinoic acids exert their negative regulation mechanisms by decreasing the ALDH1 levels at a higher concentration. Elizondo et al. (2000) showed that RARα and CCAAT/enhancer binding protein β curb ALDH1 gene reporter expression via RARα/RXRβ heterodimers. High exogenous levels of RA (1 uM) decreased ALDH1 expression and thus downregulated its activity. Apart from the interaction of CCAAT/enhancer binding protein and RARα, Elizondo et al. (2009) showed that the increased levels of RA further increased the C/EBPβ mRNA levels, which later increased GADD133 expression. Furthermore, increased levels of GADD133 decrease the DNA-binding capacity of C/EBPβ at the RAldh1 promoter, thus decreasing the ALDH1 levels. Moreb et al. (2017) showed that knockdown of ALDH1A1 or ALDH3A1 increased the resistance to 4-hydroxyperoxycyclophosphamide. However, the addition of RA at high concentrations decreases the activity of ALDH and increases the cell toxicity levels by oxidative processes.

## 3 Retinoic acids and cancer

Retinoic acid receptors are categorized into three subunits, namely, RARα, RARβ, and RARγ, which are all involved in classical retinoic acid mechanisms. Non-classical retinoid pathways are managed by rexinoic acid receptor, including RXR alpha, RXR beta, and RXR gamma. However, the designated roles of these subunits are quite contradictory to each other. RAR alpha plays a role in the proliferation of cells and is involved in the non-genomic effects. RAR beta plays an extensive role in genomic effects and mainly acts as a tumor suppressor gene. Sub-subunits of RAR beta, including β1, β2, and β3, are observed in humans. Loss of RARβ2 expression results in cancer progression and chemoresistance.

It has been shown that RAR/RXR/PPAR-mediated gene activation by retinoic acid targets the downstream activation of more than 500 genes. RAR-mediated activation highlighted cell differentiation and cell cycle arrest, whereas PPARβ/δ results in tumor proliferation (Rodriguez-Torres and Allan, 2016). The non-genomic signaling mechanism also plays a role in maintaining the anti-tumorigenic activity of retinoic acids. The retinoic acid-related orphan receptor (ROR) plays a role in decreasing colorectal cancer tumor progression by targeting the Wnt signaling mechanism in colorectal cancer-initiating cells (CCICs) (Wen et al., 2017).

### 3.1 Retinoic acids as anti-cancer drugs

Retinoic acids are mainly used as a chemotherapeutic drug for the treatment of APL. RA treatment in these patients induced the differentiation of cells unlike the proliferation due to the transfusion of the *RARA* gene (Ablain and de Thé, 2014). This led to the wide usage of ATRA for many years. Apart from APL, ATRA plays a role in reducing the cell viability and cellular proliferation in ovarian cancer cell lines. Lokman et al. (2019) showed that ATRA can inhibit proliferation and invasion in both S100A10-dependent and -independent ways.

Even though RA signaling activates hundreds of downstream targets and regulates gene function, its role in DNA repair is the least explored. With relation to BRCA1 functionality, Pin1 helps in stabilizing the protein, aiding in homologous recombination (HR)-proficient repair. The long-term use of FDA-approved PARP inhibitor monotherapy resulted in the drug resistance mechanism in breast, ovarian, pancreatic, and prostate cancer patients. Nevertheless, either the knockdown of Pin1 along with olaparib or the combination of ATRA and olaparib helped in sensitizing the TNBC tumor samples both *in vitro* and *in vivo* (Luo et al., 2020). Similarly, Chen et al. (2019) showed that co-treatment of 13 Cis-RA and 1,25-dihydroxy vitamin D3 significantly inhibited the TNF alpha-mediated cell invasion in pancreatic-derived adenocarcinoma *in vitro*. TNF alone decreased the expression of TIMP-3 and induced MMP-9, as well as cell invasion, via miR 221 upregulation. However, co-treatment upregulated TIMP-3 and reduced miR 221 upregulation by blocking JNK and NF-kB signaling, thus resulting in the decrease of cell invasion in PANC-1 and HPAF-II cell lines. Januchowski et al. (2016) proved that ATRA treatment in paclitaxel- and topotecan-resistant ovarian cancer cell lines showed a decrease in ALDH1A1 protein levels in a time-dependent manner. Their studies also confirmed that a decrease in ALDH1A1 protein levels further decreased the expression levels of p-glyco protein (encoded by ABCB1) and breast cancer resistance protein (encoded by ABCG2), thus increasing the drug sensitivity of these cell lines to chemotherapeutic drugs.

**FIGURE 4:**
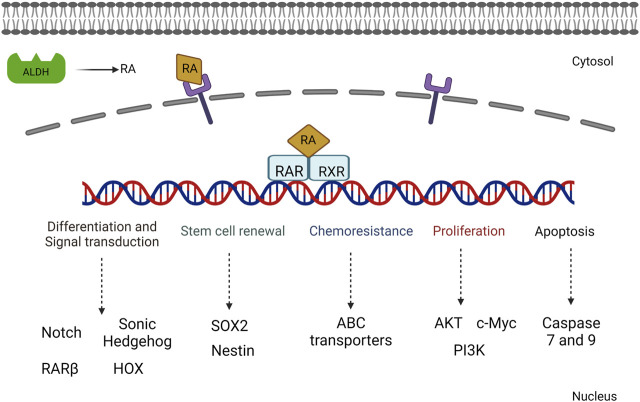
Illustration of the various signaling mechanisms activated by RA-mediated gene activation including both proliferative and cell differentiation mechanisms.

Matellan et al. (2023) showed that RAR beta expression in pancreatic targeting increased the sensitivity of overexpression of myosin light-chain 2 and reduced the stiffness of cytoskeletons. Cells treated with a RA agonist showed a decrease in the rate of invasion and cytoskeletal thickness, thus reducing the stromal microenvironment. This has shed light on developing further therapeutic strategies in pancreatic cancer (Figure 4).

### 3.2 Retinoic acid and cancer progression/their fate in cancer

On the other side of the coin, retinoic acid-mediated cancer progression has been the focus not only with respect to tumor proliferation and progression but also for acquired chemoresistance. This led to researchers focusing on various dimensions which target diverse RA-related pathways.

Retinoic acids have a potency to bind and activate both the retinoic acid/rexinoic acid receptors, which further activates the downstream signaling mechanism. However, RA can also mediate the binding and expression of orphan receptors. These RA-related orphan receptors include peroxisome proliferator-activated receptor β/δ (PPARβ/δ) family subunits, which are highly involved in the activation of pro-survival genes with respect to the high ratio of the levels of fatty acid-binding protein 5 (FABP5) to cellular retinoic acid-binding protein II (CRABPII). These orphan receptors are a part of non-canonical RA signaling which has gained popularity in cancer progression, followed by the overexpression of the aldehyde dehydrogenase 1 family. Higher concentrations of exogenous retinoic acid levels (10–50 nM) increase the levels of p-AKT in keratinocytes. High cytoplasmic levels of FABP5 are crucial to enhance the anti-apoptotic activity and ultimately results in tumor growth and development of various cancers. FABP5 expression also plays a role in preventing cell cycle arrest, followed by the continuous division of cells, resulting in tumor development (Schug et al., 2007).

Several reports have shown that retinoic acids enhance tumor cell progression in breast, lung, prostate, and ovarian cancer cell models. Retinaldehyde aldehyde dehydrogenases play a key role in synthesizing ATRA. However, aldehyde dehydrogenase 1A family subunits, including ALDH1A1 and ALDH1A3, are highly considered cancer stem cell markers, and their role in maintaining cancer stemness has been explained previously (Charafe-Jauffret et al., 2010). Ciccone et al. (2018) showed that ALDH1A1 drives tumor progression via an RAR-dependent mechanism. Their studies confirmed that breast cancer stem cells metastasize by the formation of a vasculature niche which has a subset of cancer stem cell population. ALDH1A1, being a major stem cell marker, plays a key role in tumor progression by activating hypoxia-inducing factor 1 (HIF-1) via the retinoic acid selection pathway. Chen et al. (2019) showed that RXR alpha protein expression is relatively high in pancreatic cancer patient samples, which leads to the reduction of apoptosis proteins, including Bax reduction in PANC-1 cell lines, and activation of the TGF beta signaling pathway.

### 3.3 Role of RA in activating peroxisome proliferator-activated factors

PPARs are a group of nuclear hormone receptors that function as ligand-activated transcription factors. They consist of three isoforms: PPARα, PPARβ/δ, and PPARγ. PPARs are involved in maintaining energy homeostasis by regulating glucose and lipid metabolism and transport (Lefebvre et al., 2006). They also play a role in cell proliferation, differentiation, and survival, as well as immune and inflammatory responses (Chinetti et al., 2000). Aberrant expression and function of PPARs have been observed in different types of human cancers (Smith et al., 2004; Sertznig et al., 2007). PPARα and PPARγ have been shown to inhibit tumor progression in several studies (Gao et al., 2015; Xu et al., 2016). Activation of PPARα has been associated with anti-inflammatory effects and inhibition of tumor growth in certain types of cancer. Similarly, PPARγ activation has been found to suppress cell proliferation, induce cell differentiation, and inhibit tumor invasiveness in various cancer types (Zhang et al., 2013; Hou et al., 2014). There is also evidence suggesting that PPARγ activation may have therapeutic benefits in colorectal cancer and other malignancies.

On the other hand, PPARδ has been linked to tumor development and progression. It has been implicated in the progression of colorectal cancer and associated with inflammatory bowel diseases (You et al., 2015; Zhang et al., 2017). The activation of PPARδ by specific ligands can promote inflammation and cancer growth in certain tissue types (Peters et al., 2011). It is worth noting that the effects of PPARs on tumor development can be influenced by the specific ligands that activate them. Endogenous ligands, as well as synthetic ligands, can modulate PPAR activity, and their effects may differ depending on the ligand and the tissue microenvironment.

**FIGURE 5:**
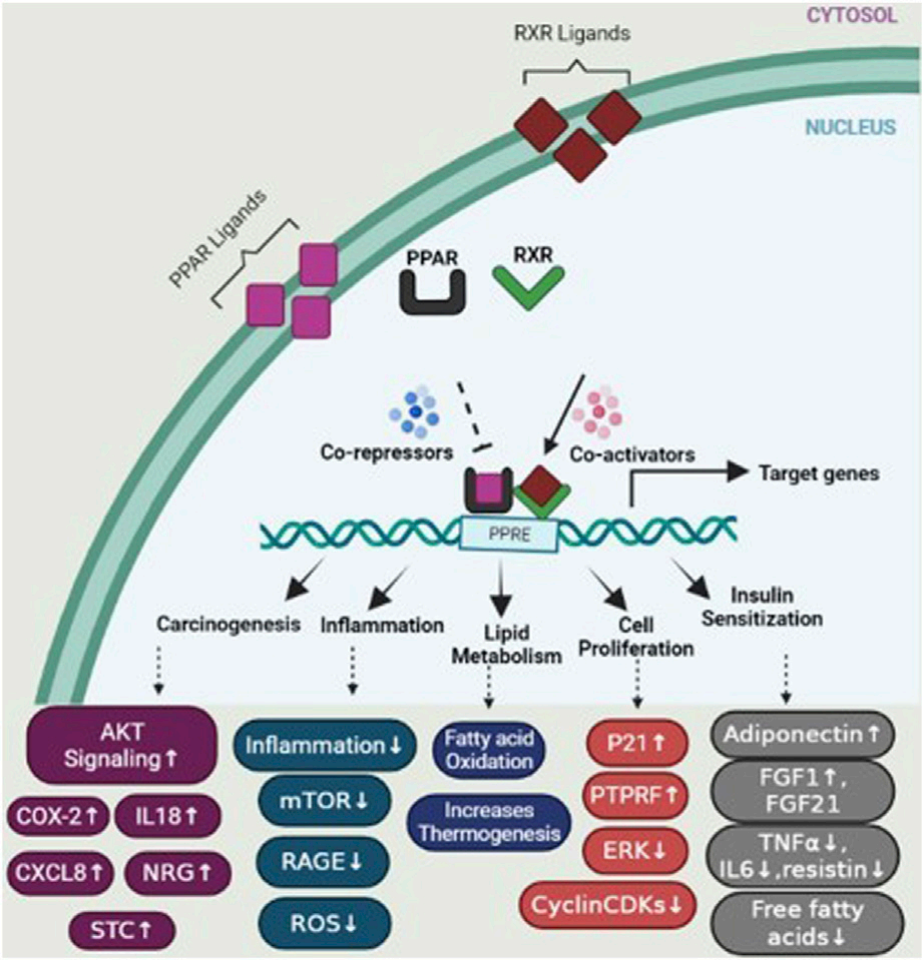
PPAR activation pathway and transcriptional regulation of target genes. Upon ligand binding, peroxisome proliferator-activated receptors (PPARs) form heterodimers with retinoid X receptors (RXRs) in the nucleus. These PPAR/RXR heterodimers interact with transcriptional coactivators and bind to specific DNA sequences known as peroxisome proliferator response elements (PPREs) located in the promoter regions of target genes, regulating the expression of genes involved in various physiological processes. In the absence of ligands, PPAR/RXR heterodimers recruit corepressors, which suppress the transcription of target genes. However, upon ligand binding, such as activation by specific agonists, the corepressors are displaced and coactivators are recruited, resulting in the activation of gene transcription. This figure also depicts several genes involved in specific mechanisms including carcinogenesis, cell proliferation, lipid metabolism, insulin sensitization, and inflammation.

Peroxisome proliferator-activated receptors (PPARs) function as transcription factors and require RXRs as heterodimeric partners to exert their regulatory function. Upon ligand binding, PPARs form heterodimers with RXRs, and together, they bind to specific DNA sequences called peroxisome proliferator response elements (PPREs) in the promoter regions of target genes (Hunsu et al., 2021). The binding of PPAR/RXR heterodimers to PPREs can lead to the activation or repression of target gene expression, depending on the specific genes involved and the ligands that bind to PPARs. Activation of PPAR/RXR heterodimers by ligands, such as fatty acids or synthetic PPAR agonists, can induce conformational changes that facilitate the recruitment of coactivator proteins and the formation of a transcriptional complex. This complex then regulates the expression of target genes involved in various cellular processes, including lipid metabolism, glucose homeostasis, inflammation, and cell differentiation (Gou et al., 2017) (Figure 5).

While ligand binding is the primary mechanism of PPAR activation, post-translational modifications such as phosphorylation can modulate PPAR activity and function. However, it is worth noting that the ligand-independent phosphorylation-mediated activation of PPARs is relatively less studied and less understood than ligand-dependent activation. Ligand binding remains the primary mode of activation for PPARs, and phosphorylation events are considered secondary regulatory mechanisms that can modulate their activity in response to cellular signaling. In cancer cells, aberrant phosphorylation events may result in the accumulation of phosphorylated RXRα (p-RXRα), which can escape degradation mechanisms, leading to an excess of non-functional RXR (Yoshimura et al., 2007). This accumulation of p-RXRα can interfere with the normal function of RXRα in a dominant-negative manner, disrupting the formation of functional heterodimers, impairing the transcriptional regulation of target genes. As a consequence, this dysregulation of RXRα phosphorylation and heterodimerization can contribute to the progression and development of cancers by disrupting normal cell growth control and response to retinoid signaling.

**FIGURE 6:**
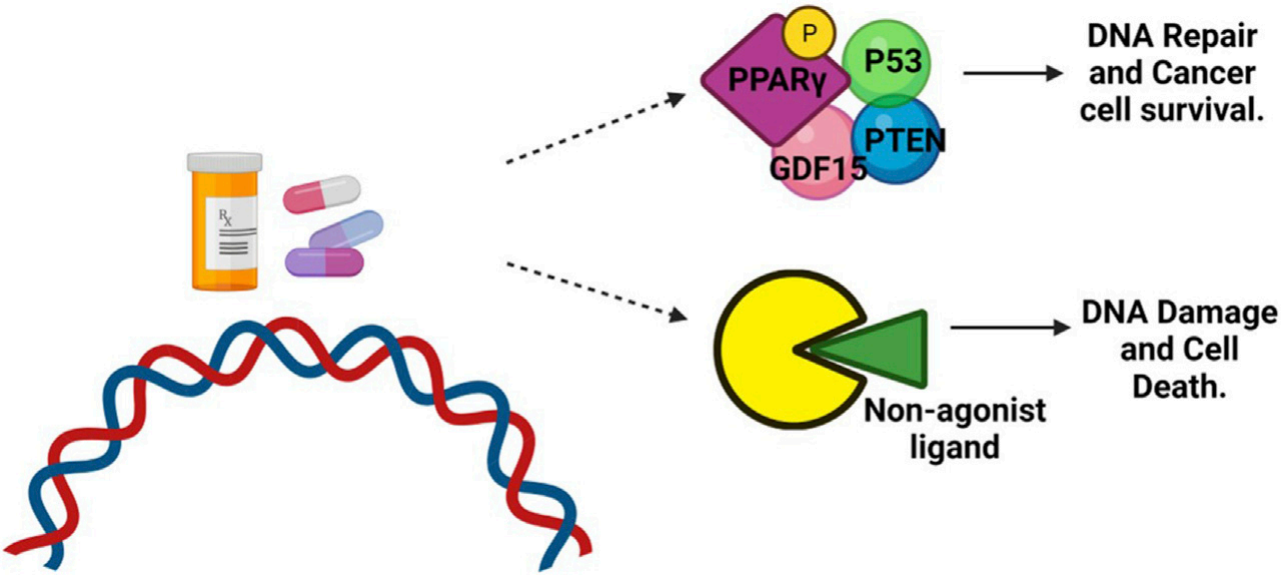
Role of PPARγ in cancer treatment. DNA-damaging agents, such as chemotherapy, can induce phosphorylation of PPARγ. This phosphorylated form of PPARγ then interacts with the tumor suppressor protein P53, antiproliferative phosphatase PTEN, and cell cycle-arresting protein GDF-15. This interaction promotes DNA repair and enhances the survival of cancer cells. However, when treated with a non-agonist PPARγ ligand, the phosphorylation of PPARγ is blocked. As a result, the interaction between PPARγ and P53 is disrupted. This disruption prevents the repair of DNA damage, leading to the accumulation of unrepaired DNA and triggering apoptotic cell death.

Clinical studies have investigated the use of PPAR agonists, particularly PPARγ ligands, in cancer treatment. However, the results from monotherapy trials have been inconclusive, suggesting limited effectiveness in advanced malignancies. Nevertheless, preclinical studies have shown that combining PPAR ligands with other agents can lead to synergistic effects in inhibiting cancer cell growth and inducing apoptosis (Yamazaki et al., 2007) (Figure 4). Combining PPAR ligands with retinoids has shown beneficial effects in preclinical studies, particularly in hematologic malignancies and solid tumors, such as breast cancer. For example, the combination of PPARγ and RXR agonists has demonstrated growth-inhibitory and differentiating effects in liposarcoma and hematologic malignancies (Demetri et al., 1999). In breast cancer, the combination of PPARγ and RXR ligands has shown promise in inhibiting aromatase expression and estrogen-dependent carcinogenesis (Burstein et al., 2003). The cooperative effects of RXR and PPARγ agonists have also been observed in colon and colorectal cancer cells (Kulke et al., 2002; Yamazaki et al., 2007). The combination treatment of oral cancer tumor cell lines with peroxisome proliferator-activated receptor gamma (PPARγ) and retinoid X receptor alpha (RXRα) agonists has shown promising results in stimulating an increase in PPARγ expression in a dose-dependent manner. This activation of the PPARγ axis leads to decreased cell proliferation, increased apoptosis, and upregulation of adipocyte differentiation markers (Rosas et al., 2020). Indeed, a significant body of evidence supports the role of PPARγ as a tumor suppressor in various types of cancer (Rosas et al., 2020). Activation of the PPARγ/RXRα signaling pathway has been shown to inhibit cell growth, decrease tumor invasiveness, and reduce the production of pro-inflammatory cytokines in different cancer types, including colon, lung, pancreatic, prostate, and breast cancers (Figure 6) (Kubota et al., 1998; Sarraf et al., 1998; Motomura et al., 2000; Bonofiglio et al., 2009).

Overall, the combined use of PPAR ligands with other agents, including retinoids, presents a potential therapeutic strategy for certain types of cancers. These combinations have shown synergistic effects in inhibiting cancer cell growth and promoting differentiation. Further research and clinical studies are needed to fully explore the efficacy and safety of these combinations in cancer treatment.

### 3.4 Role of ATRA as an immune checkpoint inhibitor

Programmed cell death 1, most popularly known as PD-1, is widely recognized for its role as a gatekeeper for immune response check. Apart from the direct gene targets to treat cancer patients, treating cancer patients with immune checkpoint inhibitors has become the focus of research over the past 5 years.

ATRA when treated as a monotherapy conventionally reduced the proliferation and metastasis of tumor cells. Furthermore, maintenance of the TME is very crucial for tumor cells, which helps in proliferation and migration. Surprisingly, Devalaraja et al. (2020) recently showed that TME induces tumor cells to produce retinoic acid, which further enhances monocyte differentiation to generate macrophages to promote tumor growth. Their studies confirmed that RA produced from tumor cells blocks dendritic cell production, which results in T-cell enrichment. Evading the immune responses via T cells is a new strategical way for tumor proliferation by producing the immune-suppressive macrophages rather than immunostimulatory dendritic cells. Their studies confirmed that suppression of Irf4 results in the blockade of dendritic cell production. Knockdown of Raldh3 via CRISPR indeed increased the immune-stimulatory antigen-presenting cells rather than macrophages. BMS493, an RAR antagonist, decreased tumor-associated macrophage production in fibro sarcoma mouse models (Devalaraja et al., 2020).

#### 3.4.1 Acquiring chemoresistance to ATRA

Chemoresistance is a conventional theory in cancer studies where anti-cancer drugs gain resistance on long-term usage. Acquiring resistance to ATRA treatment has reduced drug usage among AML, lung, and breast cancer patients. Attaining ATRA resistance can be possible in many ways, including the efflux of RA drugs via exporters, enhanced catabolism by the P450 enzyme family, decreased RAR expression due to high methylation of promotor regions, constant histone deacetylation, rearrangement of RAR/mutation in the RAR ligand-binding domain, and alterations in both co-activators and downstream targets in gene expression (Table 1).

**TABLE 1:** Overview of some of the clinical trials that used ATRA in combination with certain inhibitors to treat cancer and combat chemoresistance.

S. no.	Type of cancer	Combination and outcome	Method of study	Reference
1	Non-stem cell lung cancer/adenocarcinoma	ATRA + gefitinib reduces CSC-mediated resistance by high ALDH1A1 br/CD44 downregulation	*In vitro* using A549 and H1650	Yao et al. (2020)
2	Breast cancer	RA + FAKi decreases breast cancer cell metastasis and increases mouse survival	*In vitro* using breast cancer cell lines and *in vivo* using murine models	Castro-Guijarro et al. (2022)
3	Breast cancer	ATRA + DOX combination significantly inhibited tumor growth and synergistically inhibited cancer stem cells	*In vitro* using breast cancer cell lines and *in vivo* using orthotopic xenograft models	Sun et al. (2015)
4	NSCLC	ATRA + cisplatin combination induces differentiation	*In vitro* studies using NSCLC cell lines	Yu et al. (2020)
5	Gastric cancer	Cisplatin + ATRA combination induces apoptosis by the upregulation of miR30a	*In vitro* studies using GC spheroid models	Abbasi et al. (2022)
6	Melanoma	Allicin + ATRA shows a synergistic mechanism against CD44^+^/CD117^+^ population	*In vitro* studies using A375 cell lines	Jobani et al. (2018)
7	NSCLC	ATRA + cisplatin together reduces the ALDH + cell population and sensitizes cisplatin-resistant NSCLC cell lines	*In vitro* studies using H460, H1299, and SKMES-1	MacDonagh et al. (2021)
8	TNBC with internal mutations in NOTCH1 ICD.	ATRA + DAPT (γ-secretase inhibitor) together sensitizes the cell lines with NOTCH1 ICD, which blocks the internal aberration and constituent expression of NOTCH1, thus making the TNBC cells render sensitivity to ATRA	*In vitro* and *in vivo* studies using a large panel of TNBC cell lines	Paroni et al. (2020)

Since ATRA has been used to treat cancer, its long-term use has resulted in resistance, which further relapses the disease in most of the cancers, thus resulting in recurrence. García-Regalado et al. (2013) proved that acquired resistance to ATRA in A549 lung cancer cell lines has increased the survival and invasion of cancer cells by increasing AKT activation and upregulating the PI3K/AKT pathway via colocalizing with RARα in the cytoplasm. AKT-RARα colocalization abundantly increased AKT signaling in ATRA resistance cells. This study provided a new strategy in treating lung cancer patients by combining ATRA with AKT inhibitors, which provided an insight into clinical therapy. In prostate cancer, RA initially reduces tumor cell proliferation; however, acquiring resistance to RA has been a major concern during phase II clinical trials (Trump et al., 1997). In APL, currently, ATRA, along with arsenic trioxide with/without chemotherapy, is used to treat patients (Chen et al., 2021; Kutny et al., 2022).

#### 3.4.2 Aldehyde dehydrogenases as the targets for retinoic acid sensitivity

**FIGURE 7:**
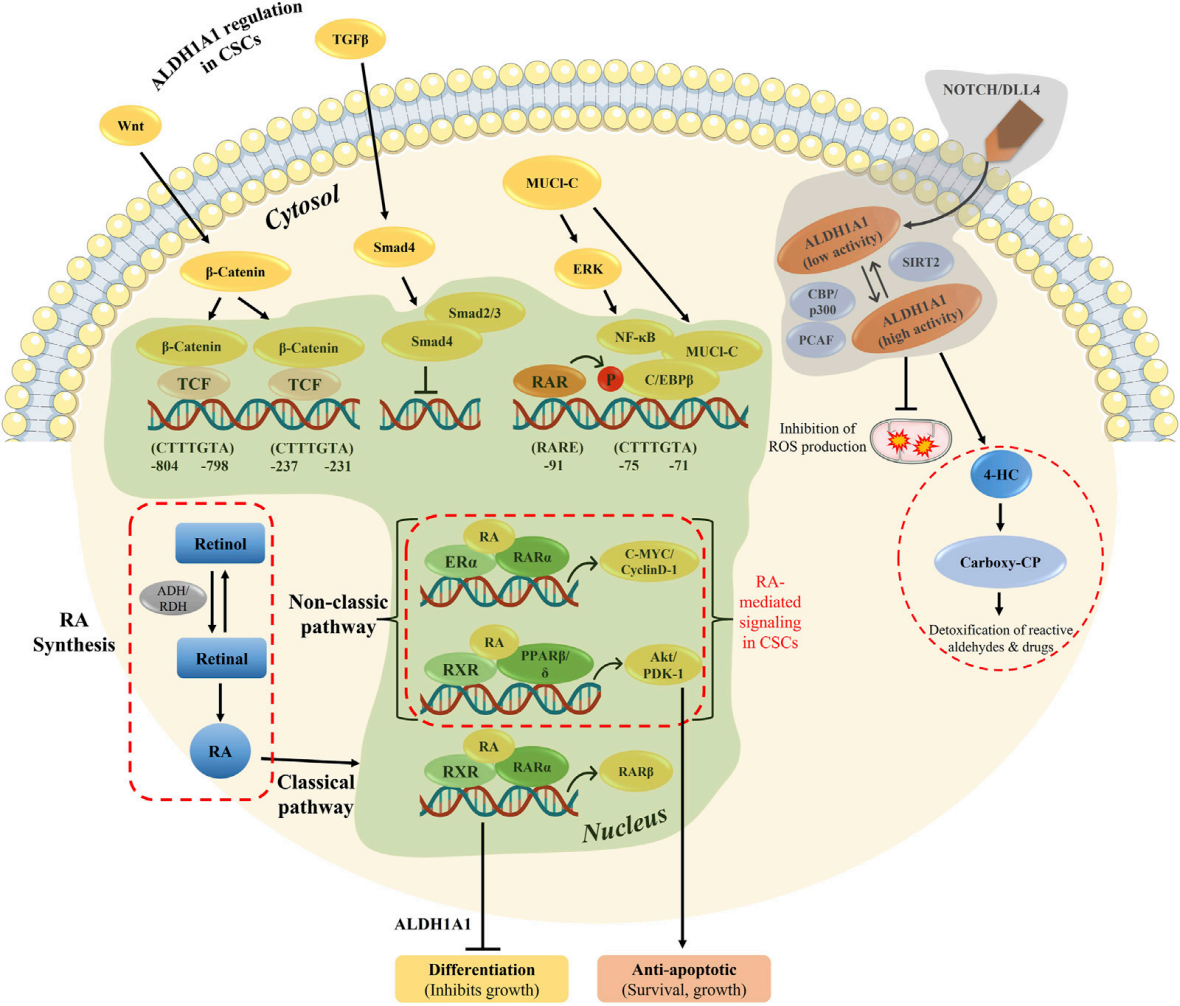
Overview of RA-mediated gene regulation in both classical and non-classical pathways, along with ALDH1A1 regulation in CSCs. The genomic and non-genomic pathways by ATRA were explained previously, which either results in cell differentiation or cellular proliferation.

Tumor progression and recurrence together severely dampen the chances of overall survival of patients. The ALDH1 family plays a prominent role in converting retinaldehyde into retinoic acid, further activating the genes in downstream activation. In addition, the ALDH family also plays a key role in cancer stem cell maintenance and drug resistance (Figure 7). Therefore, dual targeting is necessary to improve the current chemotherapeutic strategies to overcome recurrence and in enhancing the overall patient survival.

Aldehyde dehydrogenases play a role in cancer cell resistance, EMT upregulation, and their proliferation. Hence, inhibiting aldehyde dehydrogenases using their specific inhibitors, along with RAR antagonists, brings back the sensitivity to the drug. Although ALDH inhibitors have a potential to diminish ALDH1A1 expression within the cell, ATRA treatment can increase the tumor proliferation potential. A high number of genes have RARA putative sequences within themselves, and thus, ATRA treatment enhances the proliferation capacity, resulting in cancer development. Namekawa et al. (2020) showed that tubulin beta 3 class III (TUBB3) protein expression is positively correlated with tumor progression and cancer spheroid proliferation. ALDH1A1 overexpression in bladder cancer patients activates the retinoic acid signaling mechanism, thus elevating cancer progression.

Knowing that the ALDH-RA axis has a high potential for targeting cancer proliferation and enhancing a better response to chemotherapy treatments, more research is required to improve current therapeutic strategies. Biswas et al. (2022) confirmed that targeting of the SA1009-ALDH-RA axis brings back the sensitivity and reduces the tumor metastatic rate in patients with brain relapse. Their studies proved that RA upregulation by ALDH takes place via upregulation of SA1009. Targeting any of these targets brings back the sensitivity to osimertinib in EGFR-mutated lung cancer patients. Lavudi et al. (2023) also proved that targeting of ALDH1A1-RA-Pol theta has a potential in sensitizing the BRCA-mutated ovarian cancer and improves the efficacy of PARPi and increases the overall patient survival. This study used the patient-derived organoid models to show the inhibitory role of ALDH1A1 in bringing back the tumor sensitivity.

### 3.5 Clinical trials using ATRA

Being a key player for over 60 years, ATRA has undergone several clinical trials over the past years. However, due to its resistance or because of other specific competitive drug availability in various cancers, ATRA usage has been reduced in due time. Repurposing the use of ATRA as an anti-cancer regimen is widely increasing (Table 2). Kocher et al. (2020) used ATRA in their phase I clinical trials, along with gemcitabine–nab-paclitaxel, in a two-step adaptive trial design in advanced pancreatic cancer patients. Their study confirmed the stromal normalization of PDAC with ATRA as one of the combinational drug treatments showed benefits in the patients as a stromal targeting agent.

**TABLE 2:** Clinical trials that used ATRA in combination with other drugs.

S. no.	Type of the trial	Type of cancer/disease	Combination strategy	Country involved and trial number and status	Reference
1	Phase III clinical trial	NPM1-mutated AML	Gemtuzumab ozogamicin with/without ATRA	Germany and Austria; NCT00893399; trial has been completed	Döhner et al. (2023)
2	Phase II clinical trial	Thrombocytopenia	Danzol + with/without ATRA	China; NCT01667263	Feng et al. (2017)
3	Randomized phase II	Acute myeloid leukemia	Valproate and ATRA + decitabine	Germany; NCT00867672	Lübbert et al. (2020)
4	Phase II clinical trial	Advanced adenoid cystic carcinoma	ATRA	United States; trial number is not mentioned	Hanna et al. (2021)
5	Randomized control trial	Hepatocellular carcinoma	ATRA + oxaliplatin + 5 fluorouracil/leucovorin	China; registered on 2017 ChiCTR-IIR-17012916	Shi et al. (2019)
6	Phase I clinical trial	Acute myeloid leukemia	Dasatinib + ATRA	United States; NCT00892190	Redner et al. (2018)
7	Randomized trial	Acute promyelocytic leukemia	ATRA + arsenic trioxide (ATO) synergistically affected the disease-free survival rate	China, APL2012; Study outcome: chemotherapy-free or -replacing strategy was feasible among low- and intermediate-risk patients after induction therapy	Chen et al. (2021)
8	Non-randomized trial	Acute promyelocytic leukemia	ATRA + arsenic trioxide for APL pediatric patients	Australia, Canada, and US; NCT02339740; Study outcome: combination therapy resulted in the event-free survival of 2 years after the diagnosis	Kutny et al. (2022)
9	Phase 1 clinical trial	Relapsed/refractory AML and myelodysplasia	ATRA + tranylcypromine (TCP)	United States; NCT02273102; Study outcome: maximum tolerate dose usage let the patients gain significant clinical sensitivity	Tayari et al. (2021)

## 4 Future directions

Retinoic acids, as anti-cancer drugs, have been used for treating cancer patients over a long period of time. Lately, drug toxicity and chemoresistance are widely acknowledged in cancer treatments.

RA treatment alone is not highly effective in various types of cancer, including prostate, breast, and lung cancers. Knowing the fact that RA conservative sequences are present in approximately 500 genes, exogenous RA treatment effects might show a wide range of responses downstream. A vast range of RA-dependent mechanisms are not fully explored under normal conditions to comply with several forms of cancer.

In cancer therapeutics, retinoids, including RA, are used to promote tumor cell differentiation and sensitize tumors to drug combinations. Understanding the mechanisms of retinoid signaling can lead to the identification of novel drug targets and improve therapeutic strategies for cancer and other diseases, including immune-mediated inflammatory diseases. Overall, the functions of RA in cell differentiation, proliferation, stemness, and cancer progression are complex and multifaceted. Current research continues to shed light on the intricate mechanisms underlying the effects of RA and its potential therapeutic applications in various diseases.

The long-term use of ATRA in the treatment of cancers results in acquiring resistance. This has led to the development of combinational treatments in various cancers, including APL. One of the major causes of acquiring resistance is due to its dual role in activating both non-genomic and genomic effects. Schug et al. (2008) showed overcoming RA resistance in breast cancer by diverting RA from PPARβ/δ to RAR mechanisms. Apart from RARβ, which is involved in the cell differentiation process, RARγ, in turn, plays a role in the progression of several cancers, such as cholangiocarcinoma, hepatocellular carcinoma, and esophageal cancer. However, knockdown of RARγ has been proven to show drug resistance reversal in the aforementioned cancers (Huang et al., 2017).

Our findings highlight the RA synthesis and the role of RA as a chemo-preventive and a proliferative agent in various cancers. This study also highlights the RA mechanism of action and the epigenetic regulation in gene activation. We clearly exemplified the recent complete/ongoing pre-clinical trials using RA as a single drug or in combination with other drugs. Moreover, this review highlights the RAR-independent mechanisms which can be targeted in future and could also provide important information for identifying the respective RA targets alone or in combination with other downstream genes as opportunities to focus on developing novel drug discoveries, drug design, and in precision medicine by identifying the expression levels of individual RAR subtypes. Furthermore, exploring individual gene mutations, which are regulated by the RA mechanism, in cancer patients may shed some light on the treatment procedures, which, in turn, can shed light on precision medicine.

## Figures and Tables

**Table:** 

**RA**	Retinoic acid
**REs**	Retinyl esters
**ATRA**	All trans-retinoic acid
**RAR**	Retinoic acid receptor
**RXR**	Rexinoid receptor
**ALDH**	Aldehyde dehydrogenase
**CYP26A1**	Cytochrome P450 family 26 subfamily A member 1
**APL**	Acute promyelocytic leukemia
**RAE**	Retinol activity equivalents
**BCO1**	β-carotene 15-15^’^ oxygenase
**LART**	Lecithin:retinol acyltransferase
**ARAT**	Acyl-CoA:retinol acyltransferase
**RBP4**	Retinol-binding protein 4
**REHs**	Retinyl ester hydrolases
**TTR**	Transthyretin
**STRA6**	Signaling receptor and transporter of retinol STRA6
**RDH**	Retinol dehydrogenase
**CRABP1**	Cellular retinoic acid-binding protein 1
**4 keto RA**	4-keto-retinoic acid
**4 oxo RA**	4-oxo-retinoic acid
**18 hydroxy RA**	18-hydroxy retinoic acid
**4 hydroxy RA**	4-hydroxy retinoic acid
**SRBP**	Serum retinol-binding protein
**CRBP1**	Cellular retinol-binding protein 1
**RALDH**	Retinaldehyde
**SDR**	Short-chain dehydrogenase/reductase
**ROC**	Retinoid oxidoreductive complex
**NR**	Nuclear receptor
**NTD**	N-terminal domain
**AF1**	Activator function 1
**DBD**	DNA-binding domain
**LBD**	Ligand-binding domain
**RARE**	Retinoic acid receptor element
**DR**	Direct repetition
**HAT**	Histone acetyltransferase
**SMCC**	SRB- and MED-containing cofactor complex
**GTF TFIIH**	General transcription factor TF IIH

## References

[B1] Abbasi A. Hosseinpourfeizi M. Safaralizadeh R. (2022). All-trans retinoic acid-mediated miR-30a up-regulation suppresses autophagy and sensitizes gastric cancer cells to cisplatin. Life Sci. 307, 120884. 10.1016/j.lfs.2022.120884 35973456

[B2] Ablain J. de Thé H. (2014). Retinoic acid signaling in cancer: the parable of acute promyelocytic leukemia. Int. J. Cancer 135 (10), 2262–2272. 10.1002/ijc.29081 25130873

[B3] Altucci L. Gronemeyer H. (2001). The promise of retinoids to fight against cancer. Nat. Rev. Cancer 1 (3), 181–193. 10.1038/35106036 11902573

[B4] Amengual J. Zhang N. Kemerer M. Maeda T. Palczewski K. Von Lintig J. (2014). STRA6 is critical for cellular vitamin A uptake and homeostasis. Hum. Mol. Genet. 23 (20), 5402–5417. 10.1093/hmg/ddu258 24852372 PMC4168826

[B5] Balmer J. E. Blomhoff R. (2002). Gene expression regulation by retinoic acid. J. lipid Res. 43 (11), 1773–1808. 10.1194/jlr.r100015-jlr200 12401878

[B6] Bastien J. Rochette-Egly C. (2004). Nuclear retinoid receptors and the transcription of retinoid-target genes. Gene 328, 1–16. 10.1016/j.gene.2003.12.005 15019979

[B7] Bazewicz C. G. Dinavahi S. S. Schell T. D. Robertson G. P. (2019). Aldehyde dehydrogenase in regulatory T‐cell development, immunity and cancer. Immunology 156 (1), 47–55. 10.1111/imm.13016 30387499 PMC6283653

[B8] Belyaeva O. V. Adams M. K. Popov K. M. Kedishvili N. Y. (2019). Generation of retinaldehyde for retinoic acid biosynthesis. Biomolecules 10 (1), 5. 10.3390/biom10010005 31861321 PMC7022914

[B9] Berardi D. E. Ariza Bareño L. Amigo N. Cañonero L. Pelagatti M. D. Motter A. N. (2021). All-trans retinoic acid and protein kinase C α/β1 inhibitor combined treatment targets cancer stem cells and impairs breast tumor progression. Sci. Rep. 11 (1), 6044. 10.1038/s41598-021-85344-w 33723318 PMC7961031

[B10] Biswas A. K. Han S. Tai Y. Ma W. Coker C. Quinn S. A. (2022). Targeting S100a9-aldh1a1-retinoic acid signaling to suppress brain relapse in EGFR-mutant lung cancer. Cancer Discov. 12, 1002–1021. 10.1158/2159-8290.CD-21-0910 35078784 PMC8983473

[B11] Blaner W. S. Li Y. Brun P. J. Yuen J. J. Lee S. A. Clugston R. D. (2016). Vitamin A absorption, storage and mobilization. Subcell. Biochem. 81, 95–125. 10.1007/978-94-024-0945-1_4 27830502

[B12] Bleul T. Rühl R. Bulashevska S. Karakhanova S. Werner J. Bazhin A. V. (2015). Reduced retinoids and retinoid receptors' expression in pancreatic cancer: a link to patient survival. Mol. Carcinog. 54 (9), 870–879. 10.1002/mc.22158 24729540

[B13] Bonofiglio D. Cione E. Qi H. Pingitore A. Perri M. Catalano S. (2009). Combined low doses of PPARgamma and RXR ligands trigger an intrinsic apoptotic pathway in human breast cancer cells. Am. J. pathology 175 (3), 1270–1280. 10.2353/ajpath.2009.081078 PMC2731145 19644018

[B14] Brown G. (2023). Targeting the retinoic acid pathway to eradicate cancer stem cells. Int. J. Mol. Sci. 24 (3), 2373. 10.3390/ijms24032373 36768694 PMC9916838

[B15] Burstein H. J. Demetri G. D. Mueller E. Sarraf P. Spiegelman B. M. Winer E. P. (2003). Use of the peroxisome proliferator-activated receptor (PPAR) gamma ligand troglitazone as treatment for refractory breast cancer: a phase II study. Breast Cancer Res. Treat. 79, 391–397. 10.1023/a:1024038127156 12846423

[B16] Castro-Guijarro A. C. Vanderhoeven F. Mondaca J. M. Redondo A. L. Zoppino F. C. Fernandez-Muñoz J. M. (2022). Combination treatment of retinoic acid plus focal adhesion kinase inhibitor prevents tumor growth and breast cancer cell metastasis. Cells 11 (19), 2988. 10.3390/cells11192988 36230951 PMC9564078

[B17] Cecile R. Y. (2015). Retinoic acid signaling and mouse embryonic stem cell differentiation: cross talk between genomic and non-genomic effects of RA. Biochimica Biophysica Acta 1851 (1), 66–75. 10.1016/j.bbalip.2014.04.003 24768681

[B18] Charafe-Jauffret E. Ginestier C. Iovino F. Tarpin C. Diebel M. Esterni B. (2010). Aldehyde dehydrogenase 1–Positive cancer stem cells mediate metastasis and poor clinical outcome in inflammatory breast cancer. Clin. cancer Res. 16 (1), 45–55. 10.1158/1078-0432.CCR-09-1630 20028757 PMC2874875

[B19] Chen G. Hu M. Wang X. C. Du S. H. Cheng Z. X. Xu J. (2019). Effects of RXRα on proliferation and apoptosis of pancreatic cancer cells through TGF-β/Smad signaling pathway. Eur. Rev. Med. Pharmacol. Sci. 23 (11), 4723–4729. 10.26355/eurrev_201906_18053 31210298

[B20] Chen L. Zhu H. M. Li Y. Liu Q. F. Hu Y. Zhou J. F. (2021). Arsenic trioxide replacing or reducing chemotherapy in consolidation therapy for acute promyelocytic leukemia (APL2012 trial). Proc. Natl. Acad. Sci. 118 (6), e2020382118. 10.1073/pnas.2020382118 33495363 PMC8017727

[B21] Chen S. Shu G. Wang G. Ye J. Xu J. Huang C. (2022). HOXA1 promotes proliferation and metastasis of bladder cancer by enhancing SMAD3 transcription. Pathology-Research Pract. 239, 154141. 10.1016/j.prp.2022.154141 36228347

[B22] Chinetti G. Fruchart J. C. Staels B. (2000). Peroxisome proliferator-activated receptors (PPARs): nuclear receptors at the crossroads between lipid metabolism and inflammation. Inflamm. Res. 49 (10), 497–505. 10.1007/s000110050622 11089900

[B23] Cho K. W. Kwon H. J. Shin J. O. Lee J. M. Cho S. W. Tickle C. (2012). Retinoic acid signaling and the initiation of mammary gland development. Dev. Biol. 365 (1), 259–266. 10.1016/j.ydbio.2012.02.020 22387209

[B24] Choschzick I. Hirseland E. Cramer H. Schultz S. Leppert J. Tronnier V. (2014). Responsiveness of stem-like human glioma cells to all-trans retinoic acid and requirement of retinoic acid receptor isotypes α, β and γ. Neuroscience 279, 44–64. 10.1016/j.neuroscience.2014.07.078 25171789

[B25] Chuang L. S. Matsuo J. Douchi D. Bte Mawan N. A. Ito Y. (2023). RUNX3 in stem cell and cancer biology. Cells 12 (3), 408. 10.3390/cells12030408 36766749 PMC9913995

[B26] Ciccone V. Terzuoli E. Donnini S. Giachetti A. Morbidelli L. Ziche M. (2018). Stemness marker ALDH1A1 promotes tumor angiogenesis via retinoic acid/HIF-1α/VEGF signalling in MCF-7 breast cancer cells. J. Exp. Clin. Cancer Res. 37, 311. 10.1186/s13046-018-0975-0 30541574 PMC6291966

[B27] Collins S. J. (2002). The role of retinoids and retinoic acid receptors in normal hematopoiesis. Leukemia 16 (10), 1896–1905. 10.1038/sj.leu.2402718 12357341

[B28] Cunningham T. J. Duester G. (2015). Mechanisms of retinoic acid signalling and its roles in organ and limb development. Nat. Rev. Mol. Cell Biol. 16 (2), 110–123. 10.1038/nrm3932 25560970 PMC4636111

[B29] Demetri G. D. Fletcher C. D. Mueller E. Sarraf P. Naujoks R. Campbell N. (1999). Induction of solid tumor differentiation by the peroxisome proliferator-activated receptor-γ ligand troglitazone in patients with liposarcoma. Proc. Natl. Acad. Sci. 96 (7), 3951–3956. 10.1073/pnas.96.7.3951 10097144 PMC22401

[B30] Devalaraja S. To T. K. Folkert I. W. Natesan R. Alam M. Z. Li M. (2020). Tumor-derived retinoic acid regulates intratumoral monocyte differentiation to promote immune suppression. Cell 180 (6), 1098–1114. 10.1016/j.cell.2020.02.042 32169218 PMC7194250

[B31] Di Masi A. Leboffe L. De Marinis E. Pagano F. Cicconi L. Rochette-Egly C. (2015). Retinoic acid receptors: from molecular mechanisms to cancer therapy. Mol. aspects Med. 41, 1–115. 10.1016/j.mam.2014.12.003 25543955

[B32] Díez-del Val I. Martínez-Blázquez C. (2003). Cirugía de la obesidad mórbida: medicina basada en la evidencia. Cirugía Española. 74 (4), 185–192. 10.1016/s0009-739x(03)72221-0

[B33] Dittmer J. (2003). The biology of the Ets1 proto-oncogene. Mol. cancer 2 (1), 29–21. 10.1186/1476-4598-2-29 12971829 PMC194255

[B34] Döhner H. Weber D. Krzykalla J. Fiedler W. Kühn M. W. Schroeder T. (2023). Intensive chemotherapy with or without gemtuzumab ozogamicin in patients with NPM1-mutated acute myeloid leukaemia (AMLSG 09–09): a randomised, open-label, multicentre, phase 3 trial. Lancet Haematol. 10, e495–e509. 10.1016/S2352-3026(23)00089-3 37187198

[B35] Dong D. Ruuska S. E. Levinthal D. J. Noy N. (1999). Distinct roles for cellular retinoic acid-binding proteins I and II in regulating signaling by retinoic acid. J. Biol. Chem. 274 (34), 23695–23698. 10.1074/jbc.274.34.23695 10446126

[B36] Dong S. Chen S. J. Tweardy D. J. (2003). Cross-talk between retinoic acid and STAT3 signaling pathways in acute promyelocytic leukemia. Leukemia lymphoma 44 (12), 2023–2029. 10.1080/1042819031000116670 14959844

[B37] Elizondo G. Corchero J. Sterneck E. Gonzalez F. J. (2000). Feedback inhibition of the retinaldehyde dehydrogenase gene ALDH1 by retinoic acid through retinoic acid receptor alpha and CCAAT/enhancer-binding protein beta. J. Biol. Chem. 275 (50), 39747–39753. 10.1074/jbc.M004987200 10995752

[B38] Elizondo G. Medina-Díaz I. M. Cruz R. Gonzalez F. J. Vega L. (2009). Retinoic acid modulates retinaldehyde dehydrogenase 1 gene expression through the induction of GADD153-C/EBPbeta interaction. Biochem. Pharmacol. 77 (2), 248–257. 10.1016/j.bcp.2008.10.011 18992716 PMC2790144

[B39] Endo T. Freinkman E. de Rooij D. G. Page D. C. (2017). Periodic production of retinoic acid by meiotic and somatic cells coordinates four transitions in mouse spermatogenesis. Proc. Natl. Acad. Sci. 114 (47), E10132–E10141. 10.1073/pnas.1710837114 29109271 PMC5703301

[B40] Fan W. J. Ding H. Chen X. X. Yang L. (2021). All-trans retinoic acid potentiates antitumor efficacy of cisplatin by increasing differentiation of cancer stem-like cells in cervical cancer. Ann. Clin. Laboratory Sci. 51 (1), 22–29. 33653777

[B41] Feng F. E. Feng R. Wang M. Zhang J. M. Jiang H. Jiang Q. (2017). Oral all-trans retinoic acid plus danazol versus danazol as second-line treatment in adults with primary immune thrombocytopenia: a multicentre, randomised, open-label, phase 2 trial. Lancet Haematol. 4 (10), e487–e496. 10.1016/S2352-3026(17)30170-9 28917657

[B42] Fettig L. M. McGinn O. Finlay-Schultz J. LaBarbera D. V. Nordeen S. K. Sartorius C. A. (2017). Cross talk between progesterone receptors and retinoic acid receptors in regulation of cytokeratin 5-positive breast cancer cells. Oncogene 36 (44), 6074–6084. 10.1038/onc.2017.204 28692043 PMC5668194

[B43] Gao J. Liu Q. Xu Y. Gong X. Zhang R. Zhou C. (2015). PPARα induces cell apoptosis by destructing Bcl2. Oncotarget 6 (42), 44635–44642. 10.18632/oncotarget.5988 26556865 PMC4792581

[B44] García-Regalado A. Vargas M. García-Carrancá A. Aréchaga-Ocampo E. González-De la Rosa C. H. (2013). Activation of Akt pathway by transcription-independent mechanisms of retinoic acid promotes survival and invasion in lung cancer cells. Mol. cancer 12, 44. 10.1186/1476-4598-12-44 23693014 PMC3665688

[B45] Germain P. Iyer J. Zechel C. Gronemeyer H. (2002). Co-regulator recruitment and the mechanism of retinoic acid receptor synergy. Nature 415 (6868), 187–192. 10.1038/415187a 11805839

[B46] Giannì M. Bauer A. Garattini E. Chambon P. Rochette-Egly C. (2002). Phosphorylation by p38MAPK and recruitment of SUG-1 are required for RA-induced RARγ degradation and transactivation. EMBO J. 21 (14), 3760–3769. 10.1093/emboj/cdf374 12110588 PMC126119

[B47] Gilbert C. (2013). What is vitamin A and why do we need it? Community eye health 26 (84), 65. 24782580 PMC3936685

[B48] Glass C. K. Rosenfeld M. G. (2000). The coregulator exchange in transcriptional functions of nuclear receptors. Genes & Dev. 14 (2), 121–141. 10.1101/gad.14.2.121 10652267

[B49] Gonzalez F. Delahodde A. Kodadek T. Johnston S. A. (2002). Recruitment of a 19 S proteasome subcomplex to an activated promoter. Science 296 (5567), 548–550. 10.1126/science.1069490 11964484

[B50] Goodman D. S. (1984). Vitamin A and retinoids in health and disease. N. Engl. J. Med. 310 (16), 1023–1031. 10.1056/NEJM198404193101605 6369133

[B51] Gou Q. Gong X. Jin J. Shi J. Hou Y. (2017). Peroxisome proliferator-activated receptors (PPARs) are potential drug targets for cancer therapy. Oncotarget 8 (36), 60704–60709. 10.18632/oncotarget.19610 28948004 PMC5601172

[B52] Guo P. D. Lu X. X. Gan W. J. Li X. M. He X. S. Zhang S. (2016). RARγ downregulation contributes to colorectal tumorigenesis and metastasis by derepressing the hippo-yap pathway. Cancer Res. 76 (13), 3813–3825. 10.1158/0008-5472.CAN-15-2882 27325643

[B53] Hanna G. J. Oneill A. Cutler J. M. Flynn M. Vijaykumar T. Clark J. R. (2021). A phase II trial of all-trans retinoic acid (ATRA) in advanced adenoid cystic carcinoma. Oral Oncol. 119, 105366. 10.1016/j.oraloncology.2021.105366 34091189

[B54] Haushalter C. Asselin L. Fraulob V. Dollé P. Rhinn M. (2017). Retinoic acid controls early neurogenesis in the developing mouse cerebral cortex. Dev. Biol. 430 (1), 129–141. 10.1016/j.ydbio.2017.08.006 28790015

[B55] Herreros-Villanueva M. Er T. K. Bujanda L. (2015). Retinoic acid reduces stem cell–like features in pancreatic cancer cells. Pancreas 44 (6), 918–924. 10.1097/MPA.0000000000000373 26035122

[B56] Hosoda K. Sato M. Yanai K. (2015). Identification and characterization of human genomic binding sites for retinoic acid receptor/retinoid X receptor heterodimers. Adv. Biol. Chem. 5 (02), 58–72. 10.4236/abc.2015.52006

[B57] Hou Y. Gao J. Xu H. Xu Y. Zhang Z. Xu Q. (2014). PPARγ E3 ubiquitin ligase regulates MUC1-C oncoprotein stability. Oncogene 33 (49), 5619–5625. 10.1038/onc.2013.504 24292674

[B58] Hua S. Kittler R. White K. P. (2009). Genomic antagonism between retinoic acid and estrogen signaling in breast cancer. Cell 137 (7), 1259–1271. 10.1016/j.cell.2009.04.043 19563758 PMC3374131

[B59] Huang G. L. Song W. Zhou P. Fu Q. R. Lin C. L. Chen Q. X. (2017). Oncogenic retinoic acid receptor γ knockdown reverses multi-drug resistance of human colorectal cancer via Wnt/β-catenin pathway. Cell Cycle 16 (7), 685–692. 10.1080/15384101.2017.1295180 28272990 PMC5397258

[B60] Hunsu V. O. Facey C. O. Fields J. Z. Boman B. M. (2021). Retinoids as chemo-preventive and molecular-targeted anti-cancer therapies. Int. J. Mol. Sci. 22 (14), 7731. 10.3390/ijms22147731 34299349 PMC8304138

[B61] Institute of Medicine. Food and Nutrition Board (1998). Dietary reference intakes: thiamin R, niacin, vitamin B6, folate, vitamin B12, pantothenic acid, biotin, and choline. Washington (DC): National Academies Press. 23193625

[B62] Isoherranen N. Zhong G. (2019). Biochemical and physiological importance of the CYP26 retinoic acid hydroxylases. Pharmacol. Ther. 204, 107400. 10.1016/j.pharmthera.2019.107400 31419517 PMC6881548

[B63] Iturbide A. Ruiz Tejada Segura M. L. Noll C. Schorpp K. Rothenaigner I. Ruiz-Morales E. R. (2021). Retinoic acid signaling is critical during the totipotency window in early mammalian development. Nat. Struct. Mol. Biol. 28 (6), 521–532. 10.1038/s41594-021-00590-w 34045724 PMC8195742

[B64] Januchowski R. Wojtowicz K. Sterzyſska K. Sosiſska P. Andrzejewska M. Zawierucha P. (2016). Inhibition of ALDH1A1 activity decreases expression of drug transporters and reduces chemotherapy resistance in ovarian cancer cell lines. Int. J. Biochem. Cell Biol. 78, 248–259. 10.1016/j.biocel.2016.07.017 27443528

[B65] Jepsen K. Hermanson O. Onami T. M. Gleiberman A. S. Lunyak V. McEvilly R. J. (2000). Combinatorial roles of the nuclear receptor corepressor in transcription and development. Cell 102 (6), 753–763. 10.1016/s0092-8674(00)00064-7 11030619

[B66] Jiao X. Liu R. Huang J. Lu L. Li Z. Xu L. (2020). Cellular retinoic-acid binding protein 2 in solid tumor. Curr. Protein Peptide Sci. 21 (5), 507–516. 10.2174/1389203721666200203150721 32013828

[B67] Jin Y. Teh S. S. Lau H. L. Xiao J. Mah S. H. (2022). Retinoids as anti-cancer agents and their mechanisms of action. Am. J. Cancer Res. 12 (3), 938–960. 35411232 PMC8984900

[B68] Jobani B. M. Najafzadeh N. Mazani M. Arzanlou M. Vardin M. M. (2018). Molecular mechanism and cytotoxicity of allicin and all-trans retinoic acid against CD44+ versus CD117+ melanoma cells. Phytomedicine 48, 161–169. 10.1016/j.phymed.2018.05.013 30195874

[B69] Kanungo J. (2017). Retinoic acid signaling in P19 stem cell differentiation. Anti-Cancer Agents Med. Chem. Former. Curr. Med. Chemistry-Anti-Cancer Agents) 17 (9), 1184–1198. 10.2174/1871520616666160615065000 27306567

[B70] Kinyamu H. K. Chen J. Archer T. K. (2005). Linking the ubiquitin–proteasome pathway to chromatin remodeling/modification by nuclear receptors. J. Mol. Endocrinol. 34 (2), 281–297. 10.1677/jme.1.01680 15821097

[B71] Kocher H. M. Basu B. Froeling F. E. Sarker D. Slater S. Carlin D. (2020). Phase I clinical trial repurposing all-trans retinoic acid as a stromal targeting agent for pancreatic cancer. Nat. Commun. 11 (1), 4841. 10.1038/s41467-020-18636-w 32973176 PMC7518421

[B72] Kopf E. Plassat J. L. Vivat V. Chambon P. Rochette-Egly C. (2000). Dimerization with retinoid X receptors and phosphorylation modulate the retinoic acid-induced degradation of retinoic acid receptors alpha and gamma through the ubiquitin-proteasome pathway. J. Biol. Chem. 275 (43), 33280–33288. 10.1074/jbc.M002840200 10869350

[B73] Kubota T. Koshizuka K. Williamson E. A. Asou H. Said J. W. Holden S. (1998). Ligand for peroxisome proliferator-activated receptor γ (troglitazone) has potent antitumor effect against human prostate cancer both *in vitro* and *in vivo* . Cancer Res. 58 (15), 3344–3352. 9699665

[B74] Kulke M. H. Demetri G. D. Sharpless N. E. Ryan D. P. Shivdasani R. Clark J. S. (2002). A phase II study of troglitazone, an activator of the PPARgamma receptor, in patients with chemotherapy-resistant metastatic colorectal cancer. Cancer J. 8 (5), 395–399. 10.1097/00130404-200209000-00010 12416897

[B75] Kundu T. K. Das C. (Editors) (2022). Metabolism and epigenetic regulation: Implications in cancer (Springer Nature).

[B76] Kutny M. A. Alonzo T. A. Abla O. Rajpurkar M. Gerbing R. B. Wang Y. C. (2022). Assessment of arsenic trioxide and all-trans retinoic acid for the treatment of pediatric acute promyelocytic leukemia: a report from the children’s oncology group AAML1331 trial. JAMA Oncol. 8 (1), 79–87. 10.1001/jamaoncol.2021.5206 34762093 PMC8587220

[B77] Lavudi K. Banerjee A. Li N. Yang Y. Cai S. Bai X. (2023). ALDH1A1 promotes PARP inhibitor resistance by enhancing retinoic acid receptor-mediated DNA polymerase θ expression. NPJ Precis. Oncol. 7 (1), 66. 10.1038/s41698-023-00411-x 37429899 PMC10333219

[B78] Layton A. (2009). The use of isotretinoin in acne. Dermato-endocrinology. 1 (3), 162–169. 10.4161/derm.1.3.9364 20436884 PMC2835909

[B79] Lefebvre P. Chinetti G. Fruchart J. C. Staels B. (2006). Sorting out the roles of PPAR alpha in energy metabolism and vascular homeostasis. J. Clin. investigation 116 (3), 571–580. 10.1172/JCI27989 PMC1386122 16511589

[B80] Leid M. Kastner P. Chambon P. (1992). Multiplicity generates diversity in the retinoic acid signalling pathways. Trends Biochem. Sci. 17 (10), 427–433. 10.1016/0968-0004(92)90014-z 1333659

[B81] Li H. B. Huang G. Tu J. Lv D. M. Jin Q. L. Chen J. K. (2022). METTL14-mediated epitranscriptome modification of MN1 mRNA promote tumorigenicity and all-trans-retinoic acid resistance in osteosarcoma. EBioMedicine 82, 104142. 10.1016/j.ebiom.2022.104142 35810559 PMC9272358

[B82] Liang C. Qiao G. Liu Y. Tian L. Hui N. Li J. (2021). Overview of all-trans-retinoic acid (ATRA) and its analogues: structures, activities, and mechanisms in acute promyelocytic leukaemia. Eur. J. Med. Chem. 220, 113451. 10.1016/j.ejmech.2021.113451 33895500

[B83] Liu X. Nugoli M. Laferrière J. Saleh S. M. Rodrigue-Gervais I. G. Saleh M. (2011). Stromal retinoic acid receptor β promotes mammary gland tumorigenesis. Proc. Natl. Acad. Sci. 108 (2), 774–779. 10.1073/pnas.1011845108 21187396 PMC3021047

[B84] Lokman N. A. Ho R. Gunasegaran K. Bonner W. M. Oehler M. K. Ricciardelli C. (2019). Anti-tumour effects of all-trans retinoid acid on serous ovarian cancer. J. Exp. Clin. Cancer Res. 38, 10–12. 10.1186/s13046-018-1017-7 30621740 PMC6325857

[B85] Lu C. S. Shieh G. S. Wang C. T. Su B. H. Su Y. C. Chen Y. C. (2017). Chemotherapeutics-induced Oct4 expression contributes to drug resistance and tumor recurrence in bladder cancer. Oncotarget 8 (19), 30844–30858. 10.18632/oncotarget.9602 27244887 PMC5458172

[B86] Lübbert M. Grishina O. Schmoor C. Schlenk R. F. Jost E. Crysandt M. (2020). Valproate and retinoic acid in combination with decitabine in elderly nonfit patients with acute myeloid leukemia: results of a multicenter, randomized, 2× 2, phase II trial. J. Clin. Oncol. 38 (3), 257–270. 10.1200/JCO.19.01053 31794324

[B87] Luo M. L. Zheng F. Chen W. Liang Z. M. Chandramouly G. Tan J. (2020). Inactivation of the prolyl isomerase Pin1 sensitizes BRCA1-proficient breast cancer to PARP inhibition. Cancer Res. 80 (14), 3033–3045. 10.1158/0008-5472.CAN-19-2739 32193285 PMC7755124

[B88] MacDonagh L. Santiago R. M. Gray S. G. Breen E. Cuffe S. Finn S. P. (2021). Exploitation of the vitamin A/retinoic acid axis depletes ALDH1-positive cancer stem cells and re-sensitises resistant non-small cell lung cancer cells to cisplatin. Transl. Oncol. 14 (4), 101025. 10.1016/j.tranon.2021.101025 33550205 PMC7868629

[B89] Malta T. M. Sokolov A. Gentles A. J. Burzykowski T. Poisson L. Weinstein J. N. (2018). Machine learning identifies stemness features associated with oncogenic dedifferentiation. Cell 173 (2), 338–354.e15. 10.1016/j.cell.2018.03.034 29625051 PMC5902191

[B90] Mangelsdorf D. J. Evans R. M. (1995). The RXR heterodimers and orphan receptors. Cell 83 (6), 841–850. 10.1016/0092-8674(95)90200-7 8521508

[B91] Marcato P. Dean C. A. Liu R. Z. Coyle K. M. Bydoun M. Wallace M. (2015). Aldehyde dehydrogenase 1A3 influences breast cancer progression via differential retinoic acid signaling. Mol. Oncol. 9 (1), 17–31. 10.1016/j.molonc.2014.07.010 25106087 PMC5528683

[B92] Masetti R. Vendemini F. Zama D. Biagi C. Gasperini P. Pession A. (2012). All-trans retinoic acid in the treatment of pediatric acute promyelocytic leukemia. Expert Rev. anticancer Ther. 12 (9), 1191–1204. 10.1586/era.12.101 23098119

[B93] Matellan C. Lachowski D. Cortes E. Chiam K. N. Krstic A. Thorpe S. D. (2023). Retinoic acid receptor β modulates mechanosensing and invasion in pancreatic cancer cells via myosin light chain 2. Oncogenesis 12 (1), 23. 10.1038/s41389-023-00467-1 37130839 PMC10154384

[B94] McEldrew E. P. Lopez M. J. Milstein H. (2023). “Vitamin A,” in StatPearls (Treasure Island (FL): StatPearls Publishing). 29493984

[B95] Mezquita B. Mezquita P. Pau M. Gasa L. Navarro L. Samitier M. (2018). All-trans-retinoic acid activates the pro-invasive Src-YAP-Interleukin 6 axis in triple-negative MDA-MB-231 breast cancer cells while cerivastatin reverses this action. Sci. Rep. 8 (1), 7047. 10.1038/s41598-018-25526-1 29728589 PMC5935706

[B96] Mishra S. Kelly K. K. Rumian N. L. Siegenthaler J. A. (2018). Retinoic acid is required for neural stem and progenitor cell proliferation in the adult hippocampus. Stem Cell Rep. 10 (6), 1705–1720. 10.1016/j.stemcr.2018.04.024 PMC5993652 29805108

[B97] Moreb J. S. Ucar-Bilyeu D. A. Khan A. (2017). Use of retinoic acid/aldehyde dehydrogenase pathway as potential targeted therapy against cancer stem cells. Cancer Chemother. Pharmacol. 79, 295–301. 10.1007/s00280-016-3213-5 27942929

[B98] Mosher K. I. Schaffer D. V. (2018). Proliferation versus differentiation: redefining retinoic acid’s role. Stem Cell Rep. 10 (6), 1673–1675. 10.1016/j.stemcr.2018.05.011 PMC6117460 29874625

[B99] Motomura W. Okumura T. Takahashi N. Obara T. Kohgo Y. (2000). Activation of peroxisome proliferator-activated receptor γ by troglitazone inhibits cell growth through the increase of p27Kip1 in human pancreatic carcinoma cells. Cancer Res. 60 (19), 5558–5564. 11034103

[B100] Namekawa T. Ikeda K. Horie‐Inoue K. Suzuki T. Okamoto K. Ichikawa T. (2020). ALDH1A1 in patient‐derived bladder cancer spheroids activates retinoic acid signaling leading to TUBB3 overexpression and tumor progression. Int. J. Cancer 146 (4), 1099–1113. 10.1002/ijc.32505 31187490

[B101] Napoli J. L. Boerman M. H. Chai X. Zhai Y. Fiorella P. D. (1995). Enzymes and binding proteins affecting retinoic acid concentrations. J. steroid Biochem. Mol. Biol. 53 (1-6), 497–502. 10.1016/0960-0760(95)00096-i 7626500

[B102] Napoli J. L. (2016). Functions of intracellular retinoid binding-proteins. Subcell. Biochem. 81, 21–76. 10.1007/978-94-024-0945-1_2 27830500 PMC5493979

[B103] Nhieu J. Lin Y. L. Wei L. N. (2022). CRABP1 in non-canonical activities of retinoic acid in health and diseases. Nutrients 14 (7), 1528. 10.3390/nu14071528 35406141 PMC9003107

[B104] Ni X. Hu G. Cai X. (2019). The success and the challenge of all-trans retinoic acid in the treatment of cancer. Crit. Rev. food Sci. Nutr. 59 (1), S71–S80. 10.1080/10408398.2018.1509201 30277803

[B105] Ozgun G. Senturk S. Erkek‐Ozhan S. (2020). Retinoic acid signaling and bladder cancer: epigenetic deregulation, therapy and beyond. Int. J. Cancer 148 (10), 2364–2374. 10.1002/ijc.33374 33128775

[B106] Paroni G. Zanetti A. Barzago M. M. Kurosaki M. Guarrera L. Fratelli M. (2020). Retinoic acid sensitivity of triple-negative breast cancer cells characterized by constitutive activation of the Notch1 pathway: the role of rarβ. Cancers 12 (10), 3027. 10.3390/cancers12103027 33081033 PMC7650753

[B107] Peters J. M. Morales J. L. Gonzalez F. J. (2011). Modulation of gastrointestinal inflammation and colorectal tumorigenesis by peroxisome proliferator-activated receptor-β/δ (PPARβ/δ). Drug Discov. Today Dis. Mech. 8 (3-4), e85–e93. 10.1016/j.ddmec.2011.11.002 22611424 PMC3352671

[B108] Porter B. A. Ortiz M. A. Bratslavsky G. Kotula L. (2019). Structure and function of the nuclear receptor superfamily and current targeted therapies of prostate cancer. Cancers 11 (12), 1852. 10.3390/cancers11121852 31771198 PMC6966469

[B109] Quintero Barceinas R. S. García-Regalado A. Aréchaga-Ocampo E. Villegas-Sepúlveda N. González-De la Rosa C. H. (2015). All-trans retinoic acid induces proliferation, survival, and migration in A549 lung cancer cells by activating the ERK signaling pathway through a transcription-independent mechanism. BioMed Res. Int. 2015, 404368. 10.1155/2015/404368 26557664 PMC4628773

[B110] Raouf A. Li V. Kola I. Watson D. K. Seth A. (2000). The Ets1 proto-oncogene is upregulated by retinoic acid: characterization of a functional retinoic acid response element in the Ets1 promoter. Oncogene 19 (15), 1969–1974. 10.1038/sj.onc.1203505 10773887

[B111] Redner R. L. Beumer J. H. Kropf P. Agha M. Boyiadzis M. Dorritie K. (2018). A phase-1 study of dasatinib plus all-trans retinoic acid in acute myeloid leukemia. Leukemia lymphoma 59 (11), 2595–2601. 10.1080/10428194.2018.1443330 29616864 PMC6201295

[B112] Rodriguez-Torres M. Allan A. L. (2016). Aldehyde dehydrogenase as a marker and functional mediator of metastasis in solid tumors. Clin. Exp. metastasis 33, 97–113. 10.1007/s10585-015-9755-9 26445849 PMC4740561

[B113] Rosas R. Buryska S. Silver R. Wuertz B. Ondrey F. (2020). Retinoids augment thiazolidinedione PPARγ activation in oral cancer cells. Anticancer Res. 40 (6), 3071–3080. 10.21873/anticanres.14288 32487601

[B114] Rossetti S. Ren M. Visconti N. Corlazzoli F. Gagliostro V. Somenzi G. (2016). Tracing anti-cancer and cancer-promoting actions of all-trans retinoic acid in breast cancer to a RARα epigenetic mechanism of mammary epithelial cell fate. Oncotarget 7 (52), 87064–87080. 10.18632/oncotarget.13500 27894085 PMC5349971

[B115] Sarkar D. Leung E. Y. Baguley B. C. Finlay G. J. Askarian-Amiri M. E. (2015). Epigenetic regulation in human melanoma: past and future. Epigenetics 10 (2), 103–121. 10.1080/15592294.2014.1003746 25587943 PMC4622872

[B116] Sarraf P. Mueller E. Jones D. King F. J. DeAngelo D. J. Partridge J. B. (1998). Differentiation and reversal of malignant changes in colon cancer through PPARgamma. Nat. Med. 4 (9), 1046–1052. 10.1038/2030 9734398

[B117] Schug T. T. Berry D. C. Shaw N. S. Travis S. N. Noy N. (2007). Opposing effects of retinoic acid on cell growth result from alternate activation of two different nuclear receptors. Cell 129 (4), 723–733. 10.1016/j.cell.2007.02.050 17512406 PMC1948722

[B118] Schug T. T. Berry D. C. Toshkov I. A. Cheng L. Nikitin A. Y. Noy N. (2008). Overcoming retinoic acid-resistance of mammary carcinomas by diverting retinoic acid from PPARbeta/delta to RAR. Proc. Natl. Acad. Sci. 105 (21), 7546–7551. 10.1073/pnas.0709981105 18495924 PMC2396692

[B119] Sertznig P. Seifert M. Tilgen W. Reichrath J. (2007). Present concepts and future outlook: function of peroxisome proliferator‐activated receptors (PPARs) for pathogenesis, progression, and therapy of cancer. J. Cell. physiology 212 (1), 1–12. 10.1002/jcp.20998 17443682

[B120] Shi J. Sun J. Liu C. Chai Z. Wang N. Zhang H. (2019). All-trans-retinoic acid (ATRA) plus oxaliplatin plus 5-fluorouracil/leucovorin (FOLFOX) versus FOLFOX alone as palliative chemotherapy in patients with advanced hepatocellular carcinoma and extrahepatic metastasis: study protocol for a randomized controlled trial. Trials 20, 245–249. 10.1186/s13063-019-3349-9 31036040 PMC6489221

[B121] Shi W. N. Cui S. X. Song Z. Y. Wang S. Q. Sun S. Y. Yu X. F. (2017). Overexpression of SphK2 contributes to ATRA resistance in colon cancer through rapid degradation of cytoplasmic RXRα by K48/K63-linked polyubiquitination. Oncotarget 8 (24), 39605–39617. 10.18632/oncotarget.17174 28465486 PMC5503636

[B122] Siddikuzzaman Guruvayoorappan C. Berlin Grace V. M. (2011). All trans retinoic acid and cancer. Immunopharmacol. Immunotoxicol. 33 (2), 241–249. 10.3109/08923973.2010.521507 20929432

[B123] Smith M. R. Manola J. Kaufman D. S. George D. Oh W. K. Mueller E. (2004). Rosiglitazone versus placebo for men with prostate carcinoma and a rising serum prostate‐specific antigen level after radical prostatectomy and/or radiation therapy. Cancer 101 (7), 1569–1574. 10.1002/cncr.20493 15468186

[B124] Stevison F. Jing J. Tripathy S. Isoherranen N. (2015). Role of retinoic acid-metabolizing cytochrome P450s, CYP26, in inflammation and cancer. Adv. Pharmacol. 74, 373–412. 10.1016/bs.apha.2015.04.006 26233912 PMC4859867

[B125] Sun R. Liu Y. Li S. Y. Shen S. Du X. J. Xu C. F. (2015). Co-delivery of all-trans-retinoic acid and doxorubicin for cancer therapy with synergistic inhibition of cancer stem cells. Biomaterials 37, 405–414. 10.1016/j.biomaterials.2014.10.018 25453968

[B126] Tagliaferri D. Mazzone P. Noviello T. M. Addeo M. Angrisano T. Del Vecchio L. (2020). Retinoic acid induces embryonic stem cells (ESCs) transition to 2 cell-like state through a coordinated expression of Dux and Duxbl1. Front. Cell Dev. Biol. 7, 385. 10.3389/fcell.2019.00385 32010697 PMC6979039

[B127] Tayari M. M. Santos H. G. Kwon D. Bradley T. J. Thomassen A. Chen C. (2021). Clinical responsiveness to all-trans retinoic acid is potentiated by LSD1 inhibition and associated with a quiescent transcriptome in myeloid malignancies. Clin. cancer Res. 27 (7), 1893–1903. 10.1158/1078-0432.CCR-20-4054 33495312 PMC8026558

[B128] Thatcher J. E. Isoherranen N. (2009). The role of CYP26 enzymes in retinoic acid clearance. Expert Opin. drug metabolism Toxicol. 5 (8), 875–886. 10.1517/17425250903032681 PMC2730205 19519282

[B129] Topletz A. R. Tripathy S. Foti R. S. Shimshoni J. A. Nelson W. L. Isoherranen N. (2015). Induction of CYP26A1 by metabolites of retinoic acid: evidence that CYP26A1 is an important enzyme in the elimination of active retinoids. Mol. Pharmacol. 87 (3), 430–441. 10.1124/mol.114.096784 25492813 PMC4352583

[B130] Trump D. L. Smith D. C. Stiff D. Adedoyin A. Day R. Bahnson R. R. (1997). A phase II trial of all-trans-retinoic acid in hormone-refractory prostate cancer: a clinical trial with detailed pharmacokinetic analysis. Cancer Chemother. Pharmacol. 39, 349–356. 10.1007/s002800050582 9025776

[B131] Urvalek A. M. Gudas L. J. (2014). Retinoic acid and histone deacetylases regulate epigenetic changes in embryonic stem cells. J. Biol. Chem. 289 (28), 19519–19530. 10.1074/jbc.M114.556555 24821725 PMC4094062

[B132] Varkey B. (2021). Principles of clinical ethics and their application to practice. Med. Princ. Pract. 30 (1), 17–28. 10.1159/000509119 32498071 PMC7923912

[B133] Veech R. L. Eggleston L. V. Krebs H. A. (1969). The redox state of free nicotinamide–adenine dinucleotide phosphate in the cytoplasm of rat liver. Biochem. J. 115 (4), 609–619. 10.1042/bj1150609a 4391039 PMC1185185

[B134] Wang S. Wang X. Ma L. Lin X. Zhang D. Li Z. (2016). Retinoic acid is sufficient for the *in vitro* induction of mouse spermatocytes. Stem Cell Rep. 7 (1), 80–94. 10.1016/j.stemcr.2016.05.013 PMC4944723 27346680

[B135] Weeda G. Rossignol M. Fraser R. A. Winkler G. S. Vermeulen W. Van't Veer L. J. (1997). The XPB subunit of repair/transcription factor TFIIH directly interacts with SUG1, a subunit of the 26S proteasome and putative transcription factor. Nucleic acids Res. 25 (12), 2274–2283. 10.1093/nar/25.12.2274 9173976 PMC146752

[B136] Weikum E. R. Liu X. Ortlund E. A. (2018). The nuclear receptor superfamily: a structural perspective. Protein Sci. 27 (11), 1876–1892. 10.1002/pro.3496 30109749 PMC6201731

[B137] Wen Z. Pan T. Yang S. Liu J. Tao H. Zhao Y. (2017). Up-regulated NRIP2 in colorectal cancer initiating cells modulates the Wnt pathway by targeting RORβ. Mol. cancer 16 (1), 20–24. 10.1186/s12943-017-0590-2 28137278 PMC5282884

[B138] Willy P. J. Mangelsdorf D. J. (1998). Nuclear orphan receptors: the search for novel ligands and signaling pathways. Hormones Signal. 1, 307–358. 10.1016/B978-012312411-1/50011-1

[B139] Wongwarangkana C. Wanlapakorn N. Chansaenroj J. Poovorawan Y. (2018). Retinoic acid receptor beta promoter methylation and risk of cervical cancer. World J. virology 7 (1), 1–9. 10.5501/wjv.v7.i1.1 29468136 PMC5807892

[B140] Xu Y. Jin J. Zhang W. Zhang Z. Gao J. Liu Q. (2016). EGFR/MDM2 signaling promotes NF-κB activation via PPARγ degradation. Carcinogenesis 37 (2), 215–222. 10.1093/carcin/bgv252 26718225

[B141] Yamazaki K. Shimizu M. Okuno M. Matsushima-Nishiwaki R. Kanemura N. Araki H. (2007). Synergistic effects of RXR alpha and PPAR gamma ligands to inhibit growth in human colon cancer cells-phosphorylated RXR alpha is a critical target for colon cancer management. Gut 56 (11), 1557–1563. 10.1136/gut.2007.129858 17604322 PMC2095663

[B142] Yao W. Wang L. Huang H. Li X. Wang P. Mi K. (2020). All-trans retinoic acid reduces cancer stem cell-like cell-mediated resistance to gefitinib in NSCLC adenocarcinoma cells. BMC cancer 20 (1), 315–319. 10.1186/s12885-020-06818-0 32293355 PMC7161137

[B143] Yoshimura K. Muto Y. Shimizu M. Matsushima‐Nishiwaki R. Okuno M. Takano Y. (2007). Phosphorylated retinoid X receptor alpha loses its heterodimeric activity with retinoic acid receptor beta. Cancer Sci. 98 (12), 1868–1874. 10.1111/j.1349-7006.2007.00621.x 17900311 PMC11159768

[B144] You M. Yuan S. Shi J. Hou Y. (2015). PPARδ signaling regulates colorectal cancer. Curr. Pharm. Des. 21 (21), 2956–2959. 10.2174/1381612821666150514104035 26004416

[B145] Young M. J. Wu Y. H. Chiu W. T. Weng T. Y. Huang Y. F. Chou C. Y. (2015). All-trans retinoic acid downregulates ALDH1-mediated stemness and inhibits tumour formation in ovarian cancer cells. Carcinogenesis 36 (4), 498–507. 10.1093/carcin/bgv018 25742746

[B146] Yu S. Ao Z. Wu Y. Song L. Zhang P. Li X. (2020). ZNF300 promotes chemoresistance and aggressive behaviour in non‐small‐cell lung cancer. Cell Prolif. 53 (11), e12924. 10.1111/cpr.12924 33078469 PMC7653252

[B147] Zhang J. Gao Y. Yu M. Wu H. Ai Z. Wu Y. (2015). Retinoic acid induces embryonic stem cell differentiation by altering both encoding RNA and microRNA expression. PloS one 10 (7), e0132566. 10.1371/journal.pone.0132566 26162091 PMC4498831

[B148] Zhang W. Xu Y. Xu Q. Shi H. Shi J. Hou Y. (2017). PPARδ promotes tumor progression via activation of Glut1 and SLC1-A5 transcription. Carcinogenesis 38 (7), 748–755. 10.1093/carcin/bgx035 28419191

[B149] Zhang Z. Xu Y. Xu Q. Hou Y. (2013). PPARγ against tumors by different signaling pathways. Oncol. Res. Treat. 36 (10), 598–601. 10.1159/000355328 24107916

